# Genomic, regulatory and epigenetic mechanisms underlying duplicated gene evolution in the natural allotetraploid *Oryza minuta*

**DOI:** 10.1186/1471-2164-15-11

**Published:** 2014-01-06

**Authors:** Yi Sui, Bo Li, Jinfeng Shi, Mingsheng Chen

**Affiliations:** 1 State Key Laboratory of Plant Genomics, Institute of Genetics and Developmental Biology, Chinese Academy of Sciences, Beijing, China; 2 Current address: Institute of Crop Science, Chinese Academy of Agricultural Sciences, Beijing, China

**Keywords:** Comparative genomics, Wild rice, Allotetraploid, Gene silencing

## Abstract

**Background:**

Polyploid species contribute to *Oryza* diversity. However, the mechanisms underlying gene and genome evolution in *Oryza* polyploids remain largely unknown. The allotetraploid *Oryza minuta*, which is estimated to have formed less than one million years ago, along with its putative diploid progenitors (*O. punctata* and *O. officinalis*), are quite suitable for the study of polyploid genome evolution using a comparative genomics approach.

**Results:**

Here, we performed a comparative study of a large genomic region surrounding the *Shattering4* locus in *O. minuta*, as well as in *O. punctata* and *O. officinalis*. Duplicated genomes in *O. minuta* have maintained the diploid genome organization, except for several structural variations mediated by transposon movement. Tandem duplicated gene clusters are prevalent in the *Sh4* region, and segmental duplication followed by random deletion is illustrated to explain the gene gain-and-loss process. Both copies of most duplicated genes still persist in *O. minuta*. Molecular evolution analysis suggested that these duplicated genes are equally evolved and mostly manipulated by purifying selection. However, cDNA-SSCP analysis revealed that the expression patterns were dramatically altered between duplicated genes: nine of 29 duplicated genes exhibited expression divergence in *O. minuta.* We further detected one gene silencing event that was attributed to gene structural variation, but most gene silencing could not be related to sequence changes. We identified one case in which DNA methylation differences within promoter regions that were associated with the insertion of one *hAT* element were probably responsible for gene silencing, suggesting a potential epigenetic gene silencing pathway triggered by TE movement.

**Conclusions:**

Our study revealed both genetic and epigenetic mechanisms involved in duplicated gene silencing in the allotetraploid *O. minuta*.

## Background

Rice is one of the most important crops for human consumption, as it feeds more than half of the world’s population. To facilitate rice improvement, wild relative species in the genus *Oryza* have been employed as excellent genetic resources for rice breeding and genetic modification [1,2].

The genus *Oryza,* comprising two cultivated and approximately 22 wild species, is classified into 10 genome types, including six diploids (AA, BB, CC, EE, FF and GG) and four allotetraploids (BBCC, CCDD, HHJJ and KKLL) [3,4]. To better exploit the superior wild rice genetic resources, a robust analysis of phylogeny among *Oryza* species was performed several years ago [4]. Subsequently, efforts have focused on deciphering the evolutionary relationship among diploid *Oryza* species [5-7].

However, within the genus *Oryza,* almost one-third of rice species are considered to be allotetraploids, representing a large part of species diversity present in this genus [4]. Elucidating the evolutionary history of allotetraploids in *Oryza* is highly important for obtaining a complete understanding of the evolution of *Oryza*. Unfortunately, the potential progenitors of only a few species with the BBCC genome type have been identified [4,8]. Of these species, *O. minuta* was selected as the representative species of BBCC in the *Oryza* Map Alignment Project (OMAP) [2]. Comparative genomics resources are available, including a high quality bacterial artificial chromosome (BAC) library, BAC end sequences and a BAC-based physical map [9]. The genome donors of *O. minuta* were identified as diploid *O. punctata* (BB) and *O. officinalis* (CC), although some studies suggest that an extinct Asian BB genome carrier is the direct genome donor [10]. Several studies have deduced the molecular timing of BBCC formation, indicating that the formation of the allotetraploid occurred ~0.3 to 0.6 million years ago (Mya) [8,10,11].

Polyploidy and the consequences of duplicated genomes have been extensively studied in some model species [12-17]. However, few studies have investigated microstructural variations using a comparative genomics approach [18-20]. The evolutionary fate of duplicated genes has also been well-studied, and expression analysis has often allowed gene silencing to be detected [21-25]. However, the genetic and epigenetic regulatory pathways of gene expression divergence are still largely unknown.

*Shattering4* (*Sh4*), a major quantitative trait locus responsible for rice grain shattering, which encodes a transcription factor with an MYB3 DNA binding domain, plays an important role in the establishment of the abscission layer [26]. An amino acid substitution in the *Sh4* coding region affects the normal development of abscission between the grain and the pedicel and further reduces grain shattering. Human selection of this single substitution promoted the domestication of rice from wild species. To deepen our understanding of the evolution of *O. minuta*, we conducted a comparative genomic analysis of a genomic segment surrounding the *Sh4* locus among *O. minuta*, *O. punctata* and *O. officinalis*.

## Results

### Sequencing and annotation of the *Sh4*-orthologous regions

**Figure 1:**
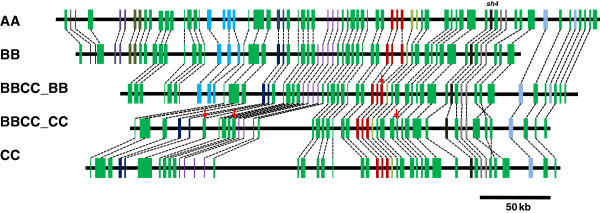
**Gene collinearity across the genus *Oryza.*** Orthologous genes in the *Sh4* regions of AA, BB, BBCC and CC were aligned with each other. The genome structure is well-conserved between each subgenome of allotetraploid and its counterpart diploid. Ten tandemly duplicated gene clusters were found within this region and four putative pseudogenes were identified. The green bars represent single copy genes in this genomic region, the black bars represent the *Sh4* gene, the other colored bars represent tandemly repeat genes and the red arrows indicate pseudogenes.

We sequenced 10 BAC clones covering the *Sh4*-orthologous regions in *O. punctata* (BB), *O. minuta* (BBCC) and *O. officinalis* (CC) (Table 1). First, we refined the gene models in *O. sativa* ssp. *japonica* cv. Nipponbare (AA). After excluding eleven retrotransposon genes and nine hypothetical genes (Additional file 1: Table S1), the remaining 70 *japonica* reference genes were used to annotate genes in the other genomes using a multiple sequence comparative approach, as described in the Methods. A total of 207 manually revised gene models in four sets of genomes (BB, CC and BBCC) were identified, including four putative pseudogenes (Additional file 1: Table S2). Comparative genomic analysis revealed that orthologous genes are well-conserved across all genomes (Figure 1).

**Table 1:** **Sequenced BAC clones from the *Sh4 *region**

**Species**	**Genome type**	**BAC clones**	**BAC length (bp)**	**Gaps**	Total sequence length (bp)*
***O. punctata***	BB	OP_Ba0062J15	161,916	2	367,482
***O. punctata***	BB	OP_Ba0087M09	208,343	3	**-**
***O. officinalis***	CC	OO_Ba0051G09	162,998	Finished	415,068
***O. officinalis***	CC	OO_Ba0021I20	256,074	3	**-**
***O. minuta *(BB)**	BBCC_BB	OM_Ba0107C17	107,440	Finished	403,493
***O. minuta *(BB)**	BBCC_BB	OM_Ba0002F21	137,910	3	**-**
***O. minuta *(BB)**	BBCC_BB	OM_Ba0232O12	184,883	1	**-**
***O. minuta *(CC)**	BBCC_CC	OM_Ba0113I02	102,513	Finished	353,205
***O. minuta *(CC)**	BBCC_CC	OM_Ba0142K17	135,139	4	**-**
***O. minuta *(CC)**	BBCC_CC	OM_Ba0186E24	123,691	1	**-**

*Total length of each genome after removing overlapping sequences.

**Figure 2:**
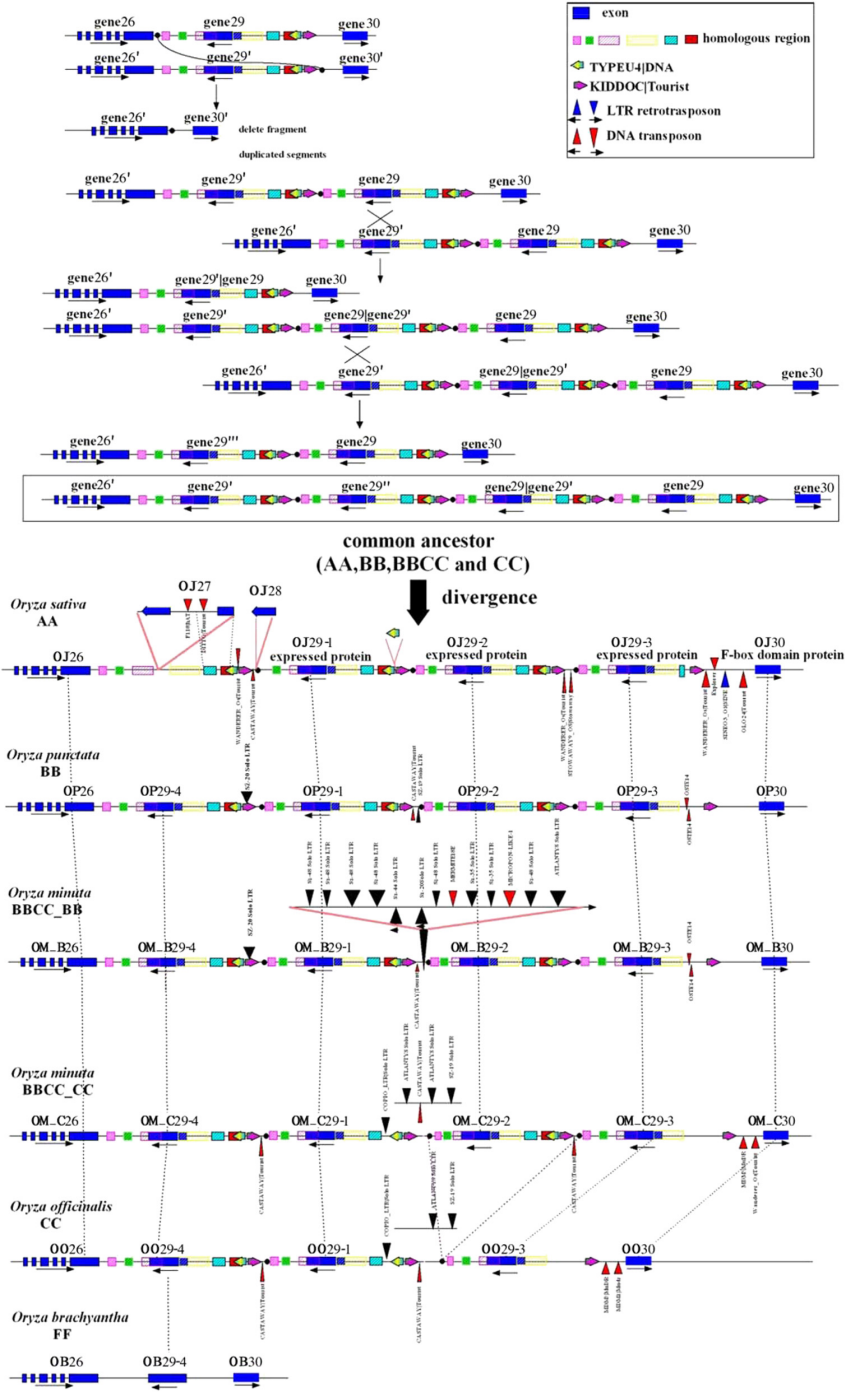
**Tandemly duplicated gene family formed through multiple rounds of segmental duplications.** Genomic segments containing Gene 29 experienced three rounds of duplication, which led to the formation of the Gene 29 tandemly duplicated gene cluster. Subsequently, gene members were randomly deleted in some species after divergence among the genus *Oryza* during this evolutionary course. Gene 29 in *O. brachyantha*, which is estimated to have separated from *Oryza sativa* ~10 MYA, still maintains the ancient copy of Gene 29, and no amplifications or deletions were detected in this genome. OJ: *O. sativa* ssp. *japonica*, OP: *O. punctata*, OM_B: BB subgenome in *O. minuta*, OM_C: CC subgenome in *O. minuta* and OO: *O. officinalis*.

Ten tandem duplicated gene clusters were identified within the *Sh4* region, and copy number changes (CNCs) were found in four of these gene clusters (16, 29, 39 and 54). Among these genes, two tandem gene families (29 and 54; represented by purple and light blue bars in Figure 1, respectively) lost one copy in the polyploid (Additional file 1: Table S2). We investigated the evolutionary mechanism of the Gene 29 cluster, where three copies of Gene 29 were present in the AA and BBCC_CC genomes, four copies were present in the BB, BBCC_BB and CC genomes and only one copy was present in the FF genome (*O. brachyantha* is the single representative of this genome type, which is the basal lineage in the genus *Oryza*). We found that multiple rounds of segmental duplication occurred in these regions to form this tandem gene cluster and they then evolved separately (Figure 2). Phylogenetic analysis indicated that Gene 29–4 is the most ancient copy and was stably maintained in the FF genome and selectively deleted in the AA genome (Additional file 1: Figure S1). Since we have no sequence information about Gene 54–2 in the BB and CC genome to ascertain whether this gene was deleted in BBCC_CC or duplicated in the other genomes, we searched for *japonica* Gene 54 by BLASTN against the *O. brachyantha* genome (FF) [11] and recently generated a draft sequence of *O. puncata* (unpublished data). We identified two tandem duplicates of Gene 54 and observed conserved gene organization in these two genomes, suggesting that this tandem duplication probably occurred before *Oryza* diversification. Phylogenetic analysis supports this notion: all 54–1 and 54–2 copies are separately clustered (Additional file 1: Figure S2). However, it cannot be confirmed whether one copy of Gene 54–2 was deleted from BC_C after polyploid formation or whether it was deleted from CC before polyploid formation, since sequence information for the CC genome is lacking. We designed 54-2-specific primers to amplify this copy in the CC genome, and no product could be detected, implying that this copy was probably deleted from the CC genome before polyploid formation (data not shown).

The details of repeat annotation are shown in Additional file 1: Table S3 and S4. We calculated and compared the TE contents for DNA type, RNA type and total (DNA and RNA) among *O. minuta* and two diploids. In BBCC_BB, the retrotransposon and total TE contents have been increasing compared with BB, while the retrotransposon and total TE contents have been decreasing in BBCC_CC compared with the CC genome (Additional file 1: Figure S3). We compared the contents of LTRs and solo LTRs between the polyploid and diploid genomes. The number of solo LTRs was much greater than that of LTRs within all genomes, which indicates that the *Oryza* genomes are experiencing contraction (Table 2).

**Table 2:** Summary of the ratio of LTRs and solo LTRs between the allotetraploid and diploids

**Pair-wise genome comparison**	**Genome specificity**	**Intact LTR-RT**	**Solo LTR**	**Intact solo LTR**	**MYA**		**Intact LTR_RT: Solo LTR (All)**
					**Range**	**Average**	
**BB VS BBCC_BB**	Only present in BBCC_BB	2	16	1	0.781-4.035	2.477	1:15
	Only present in BB	1	8	0	0.889	0.89	1:22
	Both present	0	14	2	0	0	0:14
**BBCC_CC VS CC**	Only present in BBCC_CC	1	24	0	1.723	1.723	1:60
	Only present in CC	3	24	2	0.254-5.215	1.6821	1:15
	Both present	1	36	4	0.553-1.723	1.138	1:36
**BBCC_BB VS BBCC_CC**	Only present in BBCC_BB	2	29	3	0.781-4.035	2.477	1:15
	Only present in BBCC_CC	1	59	4	1.723	1.723	1:60
	Both present	0	1	0	0	0	0:1

### Structural variation after allotetraploid formation

**Figure 3:**
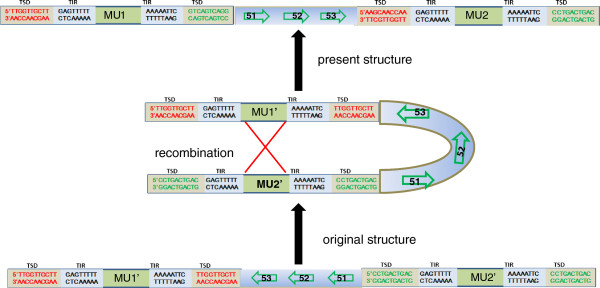
**Homologous recombination of two Mutators caused a genomic inversion.** An autonomous MuLE should have a terminal invert repeat (TIR) and 9–11 bp target site duplication (TSD). However, the TSDs of these two MuLEs (MU1 and MU2) are completely different. Sequence comparison between MU1 and MU2 indicated that two elements exchanged one side of TIR and TSD. Therefore, we propose that homologous recombination caused this inversion: Two identical MuLEs inserted into each side of these three genes (Gene 51, 52 and 53) independently. Subsequently, homologous recombination took place between the transposed enzyme coding sequences of these two MuLEs, finally leading to an inversion. MU1 and MU2 contain MuLE elements and MU1′ and MU2′ contain the original elements that were present prior to the recombination event.

Sequence comparisons revealed a ~40 kb inversion in the CC subgenome in *O. minuta* (Additional File 1: Figure S4). We identified two identical MuLE elements from two sides of this inverted segment and examined whether recombination between these two elements caused the inversion. Sequence comparisons revealed that the two elements shared identical TSD and TIR sequences, indicating that homologous recombination has occurred between these MuLEs, which indeed caused the elements to exchange TSD and TIR sequences, and thus the genomic sequence became inverted (Figure 3).

**Figure 4:**
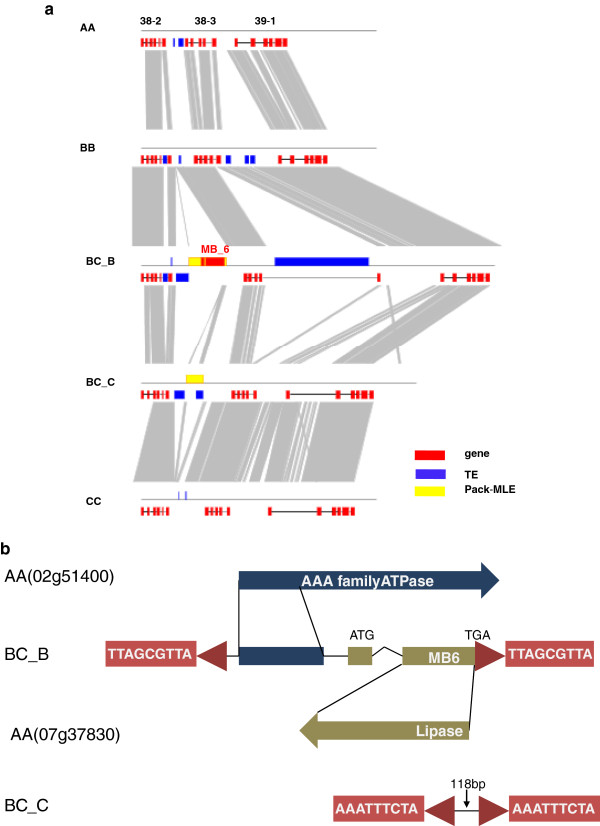
**Pack-MuLEs movements in allotetraploid *Oryza minuta *and its diploid ancestors.** Through comparative genomic analysis, we identified one Pack-MuLE specifically inserted into the BBCC_BB subgenome. FGENESH predicted one specific gene model within this MuLE element, named MB6 **(a)**. We aligned the captured sequence of this MuLE with the *japonica* reference genome and found that this MuLE probably captured sequences from multiple loci. This region (~1,100 bp) has high identity with 07 g37840 (94% identity), which produced a novel ORF by integration with some unknown sequences **(b)**. At the same genomic region of the CC subgenome in *O. minuta*, we identified the same type of MuLE with different foreign sequences (118 bp in length and lacking an exact homologous sequence in the *japonica* genome) **(a)**. These two MuLEs share a highly identical 3′ sequence and a relatively divergent 5′ sequence (probably caused by other TE insertions). The presence of completely different TSD sequences suggests that these insertion events occurred independently **(b)**.

Transposition driven by Pack-MuLEs is another factor that contributes to genome non-collinearity. We identified four transposition events that occurred in the *O. minuta* genome after polyploid formation. Notably, one Pack-MuLE in the BB subgenome captured sequence fragments from several other gene loci and became integrated into a novel predicted ORF. We failed to determine whether this ORF is functional using RT-PCR (data not shown). More interestingly, at the same region of the CC subgenomes, we identified identical type, independently inserted Pack-MuLEs with completely different captured genomic sequences (Figure 4), which indicates that these genome regions may contain preferentially inserted sequence sites for this MuLE.

### Duplicated gene evolution in *O. minuta*

To investigate duplicated gene evolution, we chose genes that were covered by BAC sequencing in all of the genomes, and therefore genes before 17 and after 53 were excluded from the following analysis (Additional file 1: Table S2). The *Sh4* regions contain 41 sets of orthologous genes (from Gene 17 to 53). Among these genes, only one deletion of Gene 29–2 was observed (represented with purple bars in Figure 1) in BBCC_CC, and both copies of the other duplicated genes were maintained. We also identified four genes that were putatively pseudogenized in the polyploid genome, which formed after polyploidy (Additional file 1: Figure S5).

We estimated the molecular evolutionary rate for duplicated genes and examined whether these genes had different rates of evolution after polyploid formation (Additional file 1: Table S5). The results from the relative rate tests suggest that most genes do not exhibit obviously different rates between the polyploid and diploids. To determine which type of selection was acting upon the duplicated genes, we calculated the ratio of non-synonymous (Ka) to synonymous (Ks) substitutions of protein coding sequences. Most duplicated genes were under purifying selection (Ka/Ks < 1), indicating that these genes are still strongly controlled after polyploidy (Additional file 1: Table S6). Similar patterns of Ks distribution between BB-BC_B and CC-BC_C also indicate that the evolutionary rates of duplicated genes were not obviously different (Additional file 1: Figure S6). We used duplicated genes to deduce the molecular timing of *O. minuta* divergence, and the results suggest that *O. minuta* was formed approximately 0.8–1.0 MYA (Additional file 1: Table S6), which is older than a previous estimate obtained by examining the *Moc1* region (~0.4 MYA) [11].

### Gene expression divergence of duplicated genes

**Figure 5:**
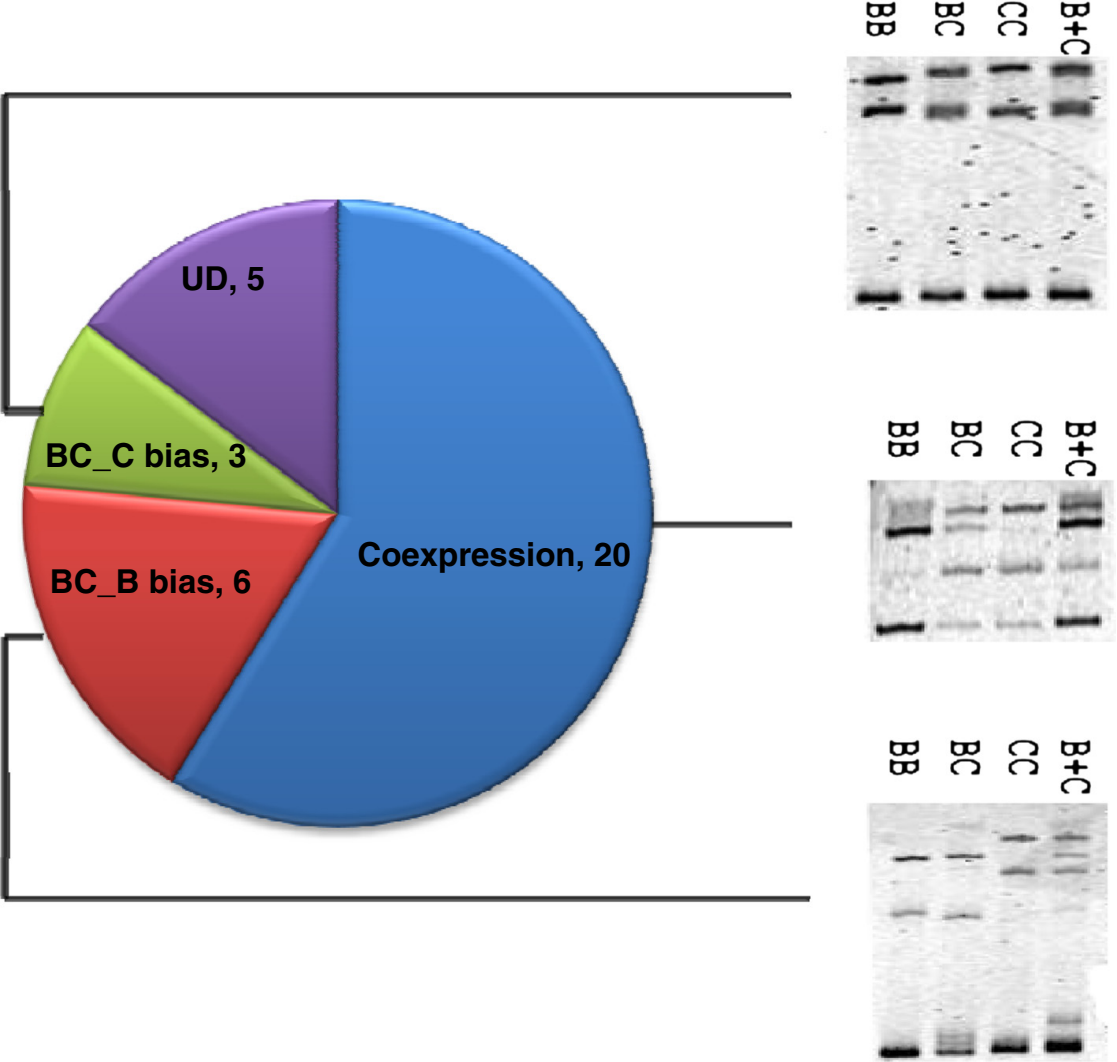
**The expression patterns of homoeologous gene pairs revealed by cDNA-SSCP analysis.** We examined a total of 34 duplicated genes to investigate the variation in gene expression patterns between the genes in each pair. The cDNA-SSCP analysis of three different types of duplicated genes, combined with PAGE, was used to reveal divergence in gene expression patterns among genes (Gene 19 represented BBCC_CC bias, Gene 43 represented BBCC_BB bias and Gene 22 represented coexpressed). Coexpressed: both copies are expressed normally; BBCC_BB bias: copy from the BB subgenome is expressed, but the one from the CC subgenome is not expressed; BBCC_CC bias: copy from the CC subgenome is expressed, but the one from the BB subgenome is not expressed.

Since duplicated genes can exhibit significant variations in gene expression, we next examined the expression divergence of 34 sets of duplicated genes in *O. minuta*. We adopted the cDNA-SSCP assay to profile changes in gene expression, and considered only qualitative variations (i.e., expressed or silencing). The cDNA-SSCP results are shown in Figure 5, which shows that approximately 31% (9/29) of the genes have one silenced copy in BBCC. The complete results are shown in Additional file 1: Figure S7 and Table S7.

To investigate whether genetic or epigenetic mechanisms regulate these genes, we first examined the genomic sequences of silenced copies of these nine genes to determine whether or how the gene structures were destroyed. The silencing in only one gene (Gene 43, whose CC subgenome copy was silenced) could be attributed to sequence variations (LTR insertion). In addition, we examined the expression patterns of two other genes (Gene 22 and Gene 26) that were identified as pseudogenes and found that both copies of the genes could be transcribed (coexpressed) (Additional file 1: Table S7). In summary, of the nine genes that exhibited expression silencing, only one was under genetic regulation. This result implies that other mechanisms, such as epigenetic regulation, may be involved in gene silencing.

**Figure 6:**
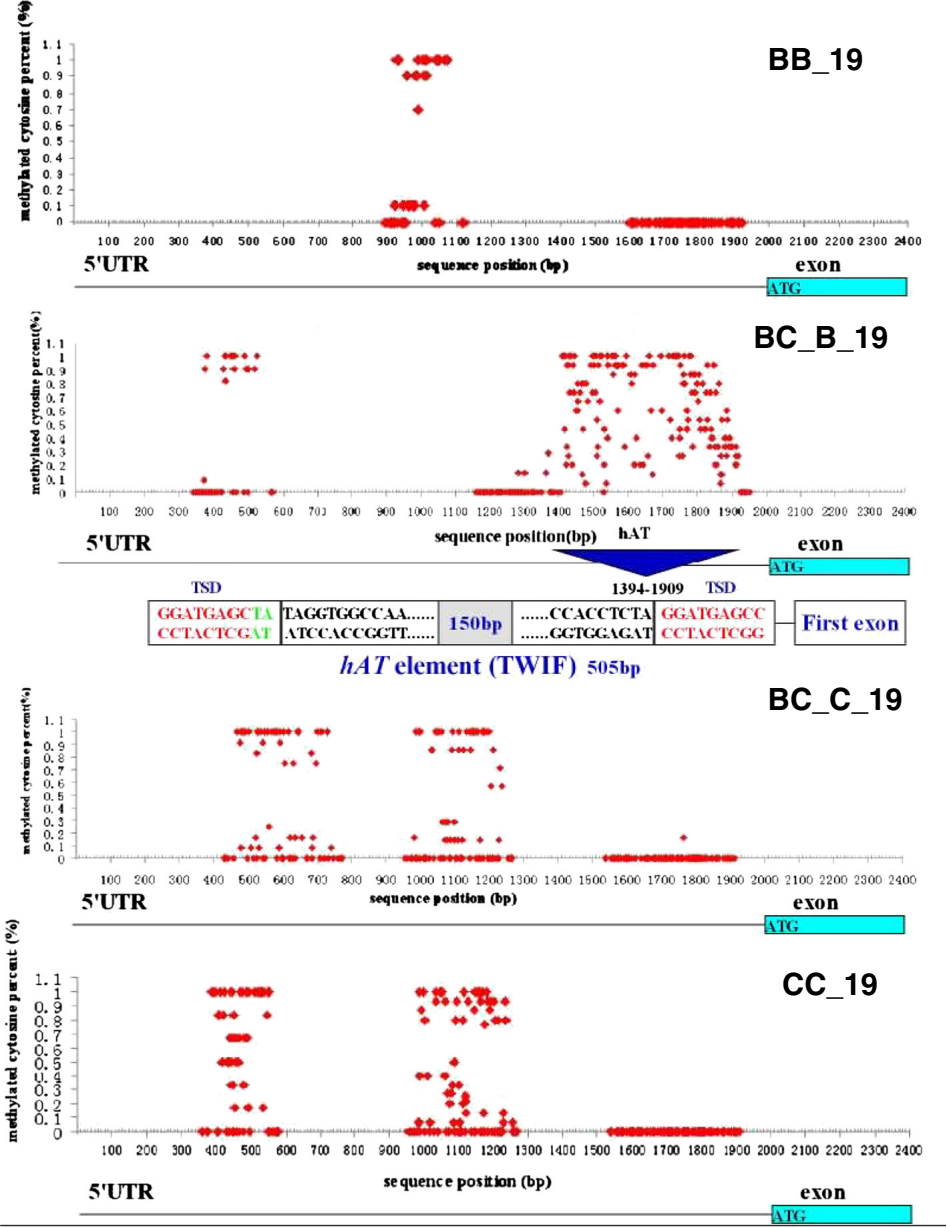
**Epigenetic pathways used to silence gene expression.** SSCP analysis revealed that Gene 19 exhibited expression divergence; one copy in the BB subgenome was silenced but no genomic variation was found to affect transcription. We examined the 5′UTRs of this gene and found a *hAT* element inserted in the predicted promoter region only in the copy in the BB subgenome. We profiled the DNA methylation pattern for the 2 kb upstream from TSS of Gene 19 in the BB subgenome and found that the DNA methylation level was dramatically increased, which inhibited normal gene expression. Using the same approach, we examined the DNA methylation levels for the BB, BC_C and CC genomes, and heavy methylation levels were not found. The red dots represent cytosines, the x-axis represents the DNA sequence (or base position) and the y-axis represents the DNA methylation level (calculated as the number of methylated Cs divided by 20). Thus, each dot represents the methylation level of each cytosine.

DNA methylation, histone modification and other epigenetic modifications can affect gene expression [27,28]. Of these regulatory mechanisms, DNA methylation is one of the most common regulator of gene expression [29]. DNA methylation profiling of whole genomes has demonstrated that the methylation levels around the transcription start sites (TSS) of genes are quite low, indicating that there is a correlation between transcription and methylation [30,31]. Thus, we selected two sets of duplicated genes to determine whether the DNA methylation levels of regulatory sequences differ between normally expressed genes and silenced genes in the polyploid. For the first gene (Gene 17), we did not observed differential methylation in the regulatory regions between the two homoelogous genes. However, for the second gene (Gene 19), we detected differential methylation profiles between the duplicated genes. A cDNA-SSCP test indicated that Gene19 is normally expressed in the CC subgenome but silenced in the BB subgenome (Additional file 1: Figure S7). Therefore, we tested the methylation levels of approximately 2 kb upstream regions from the first exon, and detected heavy methylation near the TSS region of the BB copy of the gene but almost no methylation in the CC subgenome, suggesting that this regional, heavy DNA methylation probably caused the gene silencing (Figure 6). We also examined the orthologous regions in BB and CC. No hypermethylation was found in either genome, suggesting that DNA methylation in this genomic region was not genetically inherited from the parental genomes, but it evolved after separation. We therefore examined whether the heavy methylation occurred spontaneously or whether there were other reasons for the phenomenon. When we compared the genomic sequences of this methylated region, we found that some of the sequences of BBCC_CC were completely absent from the other genomes. Annotation suggested that a *hAT* element was specifically inserted into the CC subgenome. Therefore, this heavy DNA methylation was probably brought about by TE insertion, which is in accordance with the fact that methylation is a common approach used by genomes to defend themselves against TEs [29].

## Discussion

Although several studies on synthetic and natural polyploids have provided evidence for rapid loss and gain of genomic segments (including genes) and extensive genomic reshuffling [14,16,25,32,33], sequence comparisons in the *Sh4* genomic region, combined with the results from analysis of *Adh1* and *Moc1*[8,11], suggest that the natural *Oryza* allotetraploid *O. minuta* is perhaps a relatively stable polyploid. Such stabilization is supported by the presence of conserved genes, intergenic regions and even shared TEs between polyploid and diploid genomes. However, confirming this notion would require additional investigations of other larger segments or even whole genomes.

The regulation of duplicated gene expression in polyploids has been well-studied in several model species, but few of these studies have correlated expression divergence with sequence variations [10,23,34-36]. In this study, we found that two pairs of duplicated genes annotated as pseudogenes (22 and 26) could be coexpressed in a cDNA-SSCP assay. Analysis of cDNA sequences has indicated that transcripts from pseudogenes are non-functional. Therefore, coexpression of homoelogous genes revealed by microarray analysis (or other methods) does not guarantee that both gene copies are treated equally by the genome, as mRNA sequence variations cannot be detected using these approaches. Notably, the biological significance of pseudogenes has recently been examined, especially pseudogenes that can be transcribed [37,38]. These findings suggest that pseudogenes can probably evolve from being buried in huge genomes to becoming new, functional elements, implying that pseudogenization can lead to neofunctionalization.

**Figure 7:**
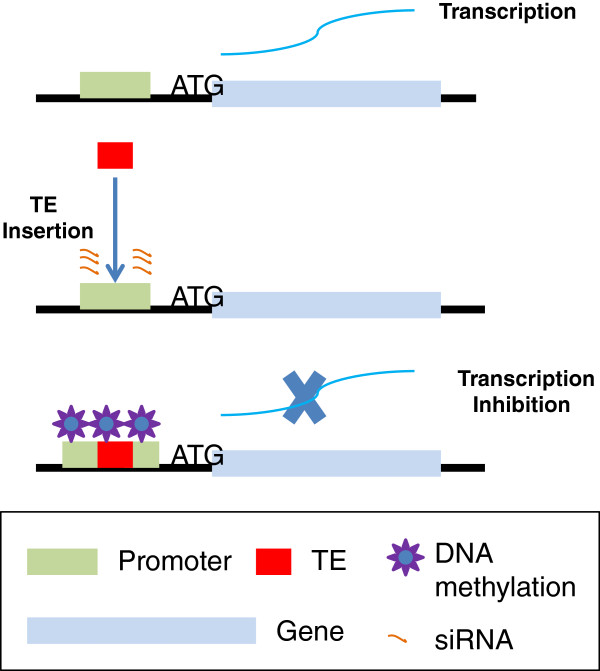
Model showing how TE-driven DNA methylation leads to silencing of duplicated genes.

Gene expression divergence was found to be more prevalent in BBCC than was previously predicted in other species [21]. Both genetic and epigenetic regulatory mechanisms were found to control duplicated genes in *O. minuta*. Deletion is a robust mechanism for controlling duplicated genes, but it is not a universal approach, as observed within the *Sh4* region, and it often occurs with multiple copy genes. Sequence analysis to detect gene functional deficiency caused by structural variation has revealed that only one gene is regulated genetically, implying that more genes may be regulated by epigenetic mechanisms. The example of TE-driven methylation silencing illustrates a potential epigenetic gene silencing pathway, which occurs as follows: after polyploidy, the presence of a duplicated genome decreases the stress upon TEs, which are then reactivated and become randomly inserted into the genome. Once integrated into sensitive genome regions (such as regulatory or translated sequences) and simultaneously supervised by the epigenetic network (e.g., DNA methylation), the function (or expression) of nearby genes may be affected to various extents and may even become completely inactivated (Figure 7). This epigenetic regulation should be more prevalent in polyploids than diploids, since it is much more difficult for this kind of high-impact insertions to escape from selection in diploids than in polyploids.

Although genome-wide experimental data are required to prove that the epigenetic pathway is the universal mechanism for gene silencing, it is currently more difficult to test epigenetic markers than genetic sequences, especially when dealing with polyploids. Nonetheless, we still propose that DNA methylation-controlled gene silencing is a prevalent mechanism, based on several observations. First, TEs have been reported to regulate gene expression for numerous genes [39,40]. For instance, a *SINE* element inserted upstream of the *FWA* coding sequence caused this gene to be epigenetically silenced in vegetative tissues of *Arabidopsis*[41]. Second, numerous TEs are likely to be inserted adjacent to genes in the BBCC genome. TEs can affect gene expression, but the effects of TEs decrease with increasing distance between the TEs and genes [42]. We investigated the distribution of several types of DNA-type TEs in the *japonica* genome. These TEs have the greatest potential to regulate nearby genes due to their preferential insertion near or within genes. Three types of TEs, including *hAT*, *Stowaway* and *Tourist*, are all present in thousands of copies (6,728, 49,810 and 40,092, respectively), and the mean distance of these TEs to genes is within 2 kb (data not shown). Therefore, we postulate that a considerable number of potential epigenetic triggers have also been buried within the BBCC genome. More importantly, according to previous reports and the results of the current study, TE insertion is not sufficient to initiate the silencing pathway; siRNA or DNA methylation is essential for initiating this program. For example, the same *hAT* elements were found in regulatory regions of the *FLC* gene in two *Arabidopsis* ecotypes, Landsberg *erecta* (Ler) and Columbia (Col). However, the roles of these two *hAT* elements in regulating *FLC* gene expression are quite different, as ~24-nt siRNA was found at higher levels in *Ler* than in *Col*; this siRNA can mediate DNA methylation and gene silencing in the *Ler* ecotype [43]. Recently, the global effects of TEs on gene expression were investigated in *Arabidopsis* and its close relative. Genome-wide analysis indicated that TEs can affect the expression of nearby genes, especially when the TEs are epigenetically modified (methylated or siRNA targeted) [42,44]. We also calculated the methylation rates of the above three types of TEs in the *japonica* genome and found that methylated copies account for over 80% of these TEs, providing further support for the potential role of silenced TEs in gene regulation. Here, we used the *japonica* genome to represent *O. minuta*. Future studies should focus on comprehensive analysis of the *O. minuta* genome to help elucidate the epigenetic regulatory pathway on a genome-wide scale.

## Conclusions

By integrating comparative genomic tools, gene expression and epigenetic analysis, our study comprehensively demonstrates how duplicated genomes and genes evolve within the S*h4* region in *O. minuta*. We found that duplicated genes are under both genetic and epigenetic regulation, and DNA methylation is proposed as a potentially important regulatory mechanism for gene silencing.

## Methods

### Plant materials and BAC library

Seeds and seedlings of *Oryza minuta* (Accession No.101141), *O. punctata* (Accession No.105690) and *O. officinalis* (Accession No.100896) were obtained from the International Rice Resource Institute (IRRI, Philippines). High-density BAC library filters and BAC clones for three *Oryza* species were purchased from the Arizona Genomics Institute (USA).

### BAC identification and sequencing

BAC clones covering the orthologous regions of the *japonica Sh4* genome segment were identified by screening *Oryza* genomic BAC libraries following the method described by Lu et al. [11]. Initial selections were conducted using two unique probes (designed with two *japonica* gene models, LOC_Os04g57350 and LOC_Os04g57600, which are located upstream and downstream of the *Shattering 4* [LOC_Os04g57530] gene locus, respectively), to hybridize to high-density filters containing three *Oryza* genomic BAC libraries. Combined with physical map positions in Finger Printed Contigs (FPC), a total of 98 positive BAC clones were identified. All screened BAC clones were digested with *Hind*III, size-selected by electrophoresis and transferred onto nylon filters for Southern blot analysis. For diploid genomes, eight additional probes were used to identify the BAC clones, which maximized the orthologous region coverage and minimized the gaps between consecutive BAC clones. To distinguish between the subgenomes of *O. minuta*, the digested map of each BAC was compared with that of *O. punctata* and *O. officinalis*. The tetrapolyploid BAC clones were divided into two groups (each from one parental genome) based on their *Hind*III digestion patterns. Ten BAC clones of three *Oryza* species were sequenced with an ABI 3730 automated sequencer (Table 1). Specifically, Sanger reads were assembled from each BAC into contigs, and BAC sequences were then merged from the same genome by identifying overlapping sequences (Table 1). Orthologous genome regions in *O. brachyantha* (FF) were recently generated [45].

### Genome annotation

A comparative gene annotation approach was taken to identify gene models in the BB, CC and BBCC genomes. Before using the *japonica Sh4* genome annotation as a reference gene model, transposon-related gene models and hypothetical genes without cDNA, ESTs and homologous proteins were excluded. Gene structures were confirmed using full-length *japonica* cDNA. To annotate non-*japonica* genomes, four wild rice genomes were initially repeat-masked and predicted using FGENESH (http://linux1.softberry.com). All predicted genes were aligned with *japonica* cDNA and proteins from rice, Sorghum, Brachypodium and Maize. Gene models without cDNA or protein supports were excluded. All gene models were manually refined based on the AA-BB-BBCC-CC multiple alignment framework. Conserved gene structures were modified based on *japonica* gene models. RT-PCR was used to detect gene structure if great variation existed between *japonica* genes and non-*japonica* genes. Multiple sequence comparisons were performed with CLUSTALW. Structural variation was detected using Artemis Comparison Tool (ACT) [46].

To identify transposon elements, RepeatMasker was initially used to annotate the TEs and other repeat sequences, followed by manual analysis (http://www.repeatmasker.org/). LTR-type transposons were also predicted with LTR_STRUC [47], LTR_Finder [48] and LTRharvest [49] to complement the results from RepeatMasker. Intact structures and other TE signatures such as target site duplication (TSD), terminal inverted repeats (TIR), polypurine tracts (PPT), primer binding sites (PBS) and long terminal repeats (LTR) were manually identified using Dotter software [50] and ACT. The insertion time of each LTR was estimated using the baseml program in PAML at a mutation rate of 1.3 × 10^-8^ per site per year [51].

Genomic structural variations, such as inversions, were also detected by ACT, followed by thorough analysis of their boundary sequences. Pack-MuLE elements were annotated manually, and to determine the captured genome sequence in the wild rice genome, the homologous sequences were searched against the *japonica* reference genome.

### Molecular evolution analysis of duplicated genes

To determine the type of selection acting upon duplicated genes, *Ka* and *Ks* values were calculated for duplicated genes in *O. minuta* using the *baseml* program with the *pairwise* model in PAML version 4.6 [52]. Alignments for coding sequences of duplicated genes were conducted with CLUSTALW [53]. Divergence times of duplicated genes were calculated with a synonymous substitution rate of 6.5 × 10^-9^ substitutions per site per year [54]. Relative evolutionary rates of duplicated genes were estimated using the *Tajima* relative rate test implemented in MEGA v5.2 [55].

### cDNA-SSCP analysis

Total RNA samples were isolated from six different tissues of three *Oryza* species, including mature leaves, mature roots, young leaves, young roots, flowers and mixed panicles. Reverse transcription was performed on mixed total RNA from all tissues, and cDNA products were used to amplify orthologous genes. SSCP analysis was then conducted with the Bio-RAD Dcode™ system following the standard protocol (Bio-RAD, USA). The SSCP results revealed the gene expression patterns of duplicated genes in *O. minuta*, including both coexpressed and gene silencing. All primer sequences are listed in Additional file 2.

### Methylation-specific PCR analysis

DNA was extracted from mature *O. punctata*, *O. officinalis* and *O. minuta* leaves, digested with restriction enzymes and treated with sodium bisulfite to convert the unmethylated cytosine residues to uracil. A set of primers was designed to amplify the genomic region of each gene from the end of the first exon to ~2 kb upstream of the gene; the size of each PCR product was approximately 150–300 bp. Primer sequences are listed in Additional file 2. The products were recovered, cloned and sequenced for DNA methylation analysis.

### Availability of supporting data

*Sh4* genome sequences for wild rices can be downloaded from NCBI at http://www.ncbi.nlm.nih.gov/nuccore/HQ827834 (BB), http://www.ncbi.nlm.nih.gov/nuccore/HQ827835 (BC_B), http://www.ncbi.nlm.nih.gov/nuccore/HQ827836 (BC_C) and http://www.ncbi.nlm.nih.gov/nuccore/HQ827837 (CC). *O. sativa* ssp. *japonica* sequences can be downloaded from http://rice.plantbiology.msu.edu/. FF genome can be downloaded from http://www.gramene.org/Oryza_brachyantha/Info/Index.

## Competing interests

The authors declare that they have no competing interests.

## Authors’ contributions

MSC designed and managed the project. YS and JFS prepared the materials and generated sequence data and performed the cDNA-SSCP analysis. YS and BL analyzed the data and performed epigenetic experiments. BL and MSC wrote the manuscript. All authors read and approved the final manuscript.

## Supplementary Material

Additional file 1: Table S1 Removed gene models from MSU Rice Genome Annotation Release 7. **Table S2.** Gene annotation results for three *Oryza* species compared with *japonica* models. **Table S3.** Annotation details of LTR retrotransposon in four *Oryza* genomes. **Table S4.** Intact elements of DNA transposons across the *Sh4* region. **Table S5.** Pair-wise relative rate test of duplicated genes between *O. minuta* and its diploid progenitors. **Table S6.** Pair-wise estimates of Ka/Ks and divergence time among the BB, CC and BBCC genomes. **Table S7.** Expression divergence of duplicated genes in allotetraploid *O. minuta.***Figure S1.** Phylogenetic analysis of Gene 29 clusters. **Figure S2.** Evolutionary analysis of Gene 54 clusters. **Figure S3.** TE contents in five *Oryza* species. **Figure S4.** Large inversion detected in the CC subgenome of *O. minuta*. **Figure S5.** Pseudogene annotation. **Figure S6.** Distributions of Ks density for homoeologous genes between two subgenomes in *O. minuta* and corresponding diploid genomes. **Figure S7.** Results of cDNA-SSCP analysis with 34 pairs of duplicated genes. Click here for file

Additional file 2 Primer information in this study. Click here for file
