# Supplementary material for: Genomic, regulatory and epigenetic mechanisms underlying duplicated gene evolution in the natural allotetraploid Oryza minuta
Source: BMC Genomics. 2014 Jan 6;15:11. doi: 10.1186/1471-2164-15-11 (PMC3890553; doi:10.1186/1471-2164-15-11)
Supplement: Additional file 1: Table S1 — Removed gene models from MSU Rice Genome Annotation Release 7. Table S2. Gene annotation results for three Oryza species compared with japonica models. Table S3. Annotation details of LTR retrotransposon in four Oryza genomes. Table S4. Intact elements of DNA transposons across the Sh4 region. Table S5. Pair-wise relative rate test of duplicated genes between O. minuta and its diploid progenitors. Table S6. Pair-wise estimates of Ka/Ks and divergence time among the BB, CC and BBCC genomes. Table S7. Expression divergence of duplicated genes in allotetraploid O. minuta.Figure S1. Phylogenetic analysis of Gene 29 clusters. Figure S2. Evolutionary analysis of Gene 54 clusters. Figure S3. TE contents in five Oryza species. Figure S4. Large inversion detected in the CC subgenome of O. minuta. Figure S5. Pseudogene annotation. Figure S6. Distributions of Ks density for homoeologous genes between two subgenomes in O. minuta and corresponding diploid genomes. Figure S7. Results of cDNA-SSCP analysis with 34 pairs of duplicated genes. [file 1471-2164-15-11-S1.pdf]

Table S1 Removed gene models from MSU Rice Genome Annotation Release 7

Code	MSU Locus	Classification	Reason to remove
**1**	LOC_Os04g56840	expressed protein	No conserved domain evidence support, No homology in other genome
**2**	LOC_Os04g56870	retrotransposon protein,putative,unclassified	(SZ-37 Copia) retrotransposon protein
**3**	LOC_Os04g56880	retrotransposon protein,putative,unclassified	(SZ-37 Copia) retrotransposon protein
**4**	LOC_Os04g56890	retrotransposon protein,putative,	(RIRE8B Gypsy) retrotransposon protein
**5**	LOC_Os04g56894	hyprothetical protein	No transcript and conserved domain evidence support, No homology in other genome
**6**	LOC_Os04g56960	hypothetical protein	No transcript and conserved domain evidence support, No homology in other genome
**7**	LOC_Os04g57040	hypothetical protein	No transcript and conserved domain evidence support, No homology in other genome
**8**	LOC_Os04g57060	retrotransposon protein, putative,unclassified	(SZ-4 Gypsy) retrotransposon protein
**9**	LOC_Os04g57100	retrotransposon protein,putative,unclassified	(SZ-33 Gypsy) retrotransposon protein
**10**	LOC_Os04g57110	retrotransposon protein,putative,unclassified	(SZ-33 Gypsy) retrotransposon protein
**11**	LOC_Os04g57154	Putative non coding sequences	No ORFs were functional
**12**	LOC_Os04g57170	hypothetical protein	No transcript and conserved domain evidence support, No homology in other genome
**13**	LOC_Os04g57240	expressed protein	Wrong cDNA, No conserved domain evidence support, No homology in other genome
**14**	LOC_Os04g57250	latency associated nuclear antigen, putative, expressed	No transcript and conserved domain evidence support, No homology in other genome
**15**	LOC_Os04g57450	retrotransposon protein, putative, unclassified	(RETRO2_LTR Gypsy) retrotransposon protein
**16**	LOC_Os04g57460	retrotransposon protein, putative, unclassified	(RETRO2_LTR Gypsy) retrotransposon protein
**17**	LOC_Os04g57470	retrotransposon protein, putative, unclassified	(RIREX_LTR Gypsy) retrotransposon protein
**18**	LOC_Os04g57570	retrotransposon protein, putative, unclassified	(SZ-55 Copia) retrotransposon protein

**19**	LOC_Os04g57580	retrotransposon protein, putative, unclassified	retrotransposon protein
**20**	LOC_Os04g57620	hyprothetical protein	No transcript and conserved domain evidence support, No homology in other genome

Table S2 Gene annotation results for three *Oryza* species compared with *japonica* models

Uniform gene name	TIGR model (V6)LOC_Os	Gene annotation					Structure variation information
		AA	BB	BC_B	BC_C	CC	
**1**	56800	1	1	NS	NS	NS	BB1 Structure variation
**2-1**	56810	2-1	2-1	NS	NS	NS	
**2-2**	56820	2-2	2-2	NS	NS	NS	BB2-2 transcript orientation reverse
**4**	56850	4	4	NS	NS	NS	
**5-1**	56900	5-1	5-1	NS	NS	NS	
**5-2**	56910	5-2	5-2	NS	NS	NS	
**6-1**	56920	6-1	6-1	NS	NS	NS	
**6-2**	56930	6-2	6-2	NS	NS	NS	
**7**	56939	7	7	NS	NS	NS	
**8**	56950	8	8	NS	NS	NS	
**9**	56970	9	9	9	NS	NS	
**10**	56980	10	10	10	NS	NS	
**11**	56990	11	11	11	NS	NS	
**12**	56995	12	12	12	NS	NS	
**13**	57010	13	13	13	NS	NS	
**14**	57020	14	14	14	NS	NS	
**15**	57030	15	15	15	NS	NS	
**16-1**	57050	16-1	16-1	16-1	NS	NS	second exon short (caused by LTR insertion)
**16-2**	57070	16-2	-	-	NS	NS	
**16-3**	57080	16-3	16-3	16-3	NS	NS	

Table S2 Continued

Uniform gene name	TIGR model (V6)LOC_Os	Gene annotation					Structure variation information
		AA	BB	BC_B	BC_C	CC	
**16-4**	57090	16-4	16-4	16-4	NS	NS	
**17**	57130	17	17	17	17	17	
**18**	57140	18	18	18	18	18	
**19**	57150	19	19	19	19	19	
**21-1**	57160	21-1	21-1	21-1	21-1	21-1	
**21-2**	57180	21-2	21-2	21-2	21-2	21-2	
**22**	57190	22	22	22	P	22	Sequence deletion
**23**	57200	23	23	23	23	23	
**24**	57210	24	24	24	24	24	
**25**	57220	25	25	25	25	25	
**26**	57230	26	26	26	P	26	premature stop codon
**29-4**	-	-	29-4	29-4	29-4	29-4	similar to AA gene 29 family
**29-1**	57260	29-1	29-1	29-1	29-1	29-1	
**29-2**	57270	29-2	29-2	29-2	-	29-2	
**29-3**	57280	29-3	29-3	29-3	29-3	29-3	
**30**	57290	30	30	30	30	30	
**31**	57300	31	31	31	31	31	
**32**	57310	32	32	32	32	32	
**33**	57320	33	33	33	33	33	
**34**	57330	34	34	34	34	34	
**35**	57340	35	35	35	35	35	
**36**	57350	36	36	36	36	36	
**37**	57360	37	37	37	37	37	
**38-1**	57370	38-1	38-1	38-1	38-1	38-1	
**38-2**	57380	38-2	38-2	38-2	38-2	38-2	
**38-3**	57390	38-3	38-3	P	38-3	38-3	Premature stop codon
**39-1**	57400	39-1	39-1	39-1	39-1	39-1	
**39-2**	57410	39-2	-	-	-	-	

Table S2 Continued

Uniform gene name	TIGR model (V6)LOC_Os	Gene annotation					Structure variation information
		AA	BB	BC_B	BC_C	CC	
**40**	57420	40	40	40	40	40	
**41**	57430	41	41	41	41	41	Rice 41: structure change (have two exon), other single exon
**42**	57440	42	42	42	42	42	
**43**	57480	43	43	43	P	43	LTR insertion
**44**	57490	44	44	44	44	44	
**45**	57500	45	45	45	45	45	
**46**	57510	46	46	46	46	46	Gap
**47**	57520	47	47	47	47	47	Gap
**48**	57530	48	48	48	48	48	
**49**	57540	49	49	49	49	49	
**50-1**	57550	50-1	50-1	50-1	50-1	50-1	
**50-2**	57560	50-2	50-2	50-2	50-2	50-2	
**51**	57590	51	51	51	51	51	
**52**	57600	52	52	52	52	52	
**53**	57610	53	53	53	53	53	
**54-1**	57630	54-1	NS	54-1	54-1	54-1	
**55**	57640	55	NS	55	55	55	
**56**	57650	56	NS	56	56	56	
**54-2**	57660	54-2	NS	54-2	-	NS	
**57**	57670	57	NS	57	57	NS	
**58**	57680	58	NS	58	NS	NS	
**59**	57690	59	NS	59	NS	NS	
**60**	57700	60	NS	NS	NS	NS	
**Total count**	**70**	**72**	**61**	**58**	**44**	**44**	

Note: "-" indicates not present; "NS" indicates no sequences were covered by BAC sequencing; "P" indicates pseudo gene; Genes 17–53 are core genes used to investigate duplicated gene evolution in our analysis. The code is not continuous because we maintained the original code for each gene model after deleting some gene models in order to not affect other analysis.

Table S3 Annotation details of LTR retrotransposon in four *Oryza* genomes

Species	Family	Name	Position and structure features	Strand	TSD(+strand 5'-3')	Ks	Divergent time（Mya）
O. sativa							
**Solo LTR**	*Gypsy*	SZ-19_LTR	9180-10762	C	GCGAT		
	Gypsy	SZ-44_LTR	11165-12552	C	CCTCC		
	Gypsy	Gypsy-A_LTR	67264-70544	C	GTGGT		
	Copia	COPI1_LTR	457506-457793	C	ATTAG		
**LTR**	*Copia*	SZ-37_LTR	Location:26713-38769 Len: 12057 Strand:- 5'-LTR: 26713-28296 Len: 1584 3'-LTR: 37186-38769 Len: 1584 5'-TG: TG , TG 3'-CA: CA , CA TSR: 26708-26712 , 38770-38774 [GCAGC] PBS: [14/17] 37164-37180 (-MetCAT) GAGACCAAGCTCTGATA PPT: [13/15] 28297-28311 ATCTCCCCCTTTTTG	C	GCAGC	0.0265	1.01923
	Gypsy	SZ-33_LTR	Location:155433-166868 Len: 11436 Strand:- 5'-LTR:155433-156008 Len: 576 3'-LTR:166296-166868 Len: 573 5'-TG :TG , TG 3'-CA :CT , CA TSR :155428-155432 , 166869-166873 [AATAT] PBS:[15/15] 166277-166291 (-ArgCCT) CCCCTACCTGGCGCG PPT:[11/15] 156053-156067 GTTGTCGTCGTTCTT Domain: 160347-160850 [possible ORF:160002-160850, (IN (core))]			0.0458	1.7615
	Gypsy	RETRO2_LTR	Location : 308064-320959 Len: 12896 Strand:- 5'-LTR:308064-309139 Len: 1076 3'-LTR:319878-320959 Len: 1082 5'-TG :TG , TG 3'-CA :CA , CA TSR :308059-308063 , 320960-320964 [AGAAC]	C	AGAAC	0.0056	0.2153

			PBS:[15/15] 319858-319872 (-ArgCCT) CCCCTACCTGGCGCG				
			PPT:[11/15] 309163-309177 GCGATTACCCTCCTC				
			Domain: 314018-314506 [possible ORF:313637-314992, (IN (core))]				
			Domain: 315038-315427 [possible ORF:314993-316279, (RNase H)]				
			Domain: 316626-317081 [possible ORF:316515-317363, (RT)]				
	Gypsy	RIREX_LTR	Location : 321446-329814 Len: 8369 Strand:-	C	CGTGG	0.0000	0
			5'-LTR:321446-321875 Len: 430 3'-LTR:329390-329814 Len: 425				
			5'-TG :TG , TG 3'-CA :CA , CA TSR :321441-321445 , 329815-329819				
			[CGTGG]				
			PBS:[15/15] 329370-329384 (-ArgCCT) CCCCTACCTGGCGCG				
			PPT:[11/15] 321909-321923 ATCTTCTCTCGAACC				
	Copia	SZ-55_LTR	Location : 385697-396005 Len: 10309 Strand:-	C	ATTGG	0.0030	0.11538
			5'-LTR:385697-387319 Len: 1623 3'-LTR:394390-396005 Len: 1616				
			5'-TG :TG , TG 3'-CA :CA , CA TSR :385692-385696 , 396006-396010				
			[ATTGG]				
			PBS:[14/15] 394370-394384 (-MetCAT)AACCGGGCTCTGATA				
			PPT:[11/15] 387320-387334ATCTCCCCCTTGATG				
			Domain: 390692-391192 [possible ORF:388625-391780, (IN (core))]				

O. punctata							
**SoloLTR**	COPIA3-LTR	*Copia*	342283..342552	C	5'_GCCTTAG		
					3'_ACCTTAG		
	ATLANTYS-LTR_OS	*Gypsy*	84424..85889	C	GAGGG		
	SZ-37_LTR	*Copia*	89863..91316		5'_GAGGT 3'_AAGGT		

**LTR**	RN10LTR	*Gypsy*	Location:63674-68867 Len:5194 Strand: 5'-LTR:63674-63935 Len:262 3'-LTR:68605-68867 Len:263 5'-TG:TG,TG 3'-CA:CA ,CA TSR:63669-63673,68868-68872 [AGATC] PPT:[12/15] 63936-63950 TTCCCCTCCGCTTGA Domain: 64550-65044 [possible ORF:63908-66004, (IN (core]	C	AGATC	0.0231	0.88846
O. minuta **(BB)**							
**SoloLTR**	ATLANTYS-LTR_OS	*Gypsy*	19929-21409		CGTGG		
	ATLANTYS-LTR_OS	*Gypsy*	21666-23133	C	TSD_GAGGG		
	SZ-37_LTR	*Copia*	24485-25964		5'_GAGGT_3'_AAGGT		
**LTR**	OSTONOR1_LTR	*Copia*	Location : 288119 - 294442 Len: 6324 Strand:- 5'-LTR : 288119 - 288572 Len: 454 3'-LTR : 293988 - 294442 Len: 455 5'-TG : TG , TG 3'-CA : CA , CA TSR : 288114 - 288118 , 294443- 294447 [TGGAC] PBS : [15/16] 293968 - 293983 (-MetCAT) PBS: ACTCCTAAACCTTGCTCTGATACCAT MetCAT: AAACCTTGCTCTGATA PPT : [14/15] 288592 - 288606 CCTTTCTTACTTCTC Domain: 290932 - 291393 [possible ORF:290929-292326, (IN (core))]	C	TGGAC	0.0203	0.780769
	SZ-54A_LTR	*Gypsy*	Location : 369974 - 375174 Len: 5201 Strand:- 5'-LTR : 369974 - 370370 Len: 397 3'-LTR : 374737 - 375174 Len: 438 5'-TG : TG , TG 3'-CA : CA , CA TSR : 369970 - 369973 , 375175- 375178 [GTAT] PBS : [14/17] 374678 - 374694 (-AsnGTT) TGCTGGTGG--TGGGGA	C	GTAT	0.1049	4.034615

			PPT : [13/15] 370371- 370385 TCTCTCCCCACCTCG<br>Domain: 371078 - 371458 [possible ORF:371075-371788, (IN (core))]<br>Domain: 372579 - 373004 [possible ORF:372366-373529, (RT)		
O. minuta **(CC)**					
**Solo LTR**	COPIO_LTR	*Copia*	91722-93161		GTGCC
	SZ-31_LTR	*Gypsy*	117166-118179		NO
	SZ-7_LTR	*Gypsy*	186783-187351		NO
	SZ-37_LTR	*Copia*	188398-189845	C	ACCGC
**LTR**	SC-3_LTR	*Copia*	Location : 210156 - 215361 Len: 5206 Strand:+<br>5'-LTR : 210156 - 210524 Len: 369 3'-LTR : 215014 - 215361 Len: 348<br>5'-TG : TG , TG 3'-CA : CA , TA TSR : 210151 - 210155 , 215362 - 215366 [GAATC]<br>PBS : [16/18] 210530 - 210547 (MetCAT) TATCAGAGCCACACTTTC<br>PPT : [12/15] 214917 - 214931 GAATGATGGAATAAG<br>Domain: 212915 - 213445 [possible ORF:212219-213514, (IN (core))]		GAATC
O. officinalis **(CC)**					
**Solo LTR**	SZ-44_LTR	*Gypsy*	42159-43518	C	TTTAT
	COPIO_LTR	*Copia*	98043-99415		5_GTGTC_3_GTGCC
	ATLANTYS-LTR_OS	*Gypsy*	101401-102980		AGTTA
	SZ-33_LTR	*Gypsy*	185108-185704		NO

	SZ-7_LTR	*Gypsy*	263762-264330		5_ATCCT_3_ACCCT		
	SZ-37_LTR	*Copia*	265256-266675	C	ACCGC		
**LTR**	SC-3_LTR	*Copia*	Location : 66128 - 71917 Len: 5790 Strand:+<br>5'-LTR : 66128 - 66431 Len: 304 3'-LTR : 71569 - 71917 Len: 349<br>5'-TG : TG , TG 3'-CA : CA , CA TSR : 66123 - 66127 , 71918 - 71922 [GATCA]<br>PBS : [16/21] 66452 - 66472 (LysTTT) CGTCGTCTTGTGGGACAATTG<br>PPT : [14/15] 71510 - 71524 AGGGAAGGGGTGGAA		GATCA	0.0144	0.553846
	SZ_LTR	*Gypsy*	Location : 174363 - 184310 Len: 9948 Strand:-<br>5'-LTR : 174363 - 175311 Len: 949<br>3'-LTR : 183360 - 184310 Len: 951<br>5'-TG : TG , TG<br>3'-CA : CA , CA<br>TSR : 174358 - 174362 , 184311 - 184315 [CCATC]<br>PBS : [17/18] 183337 - 183354 (-ArgCCT)<br>PPT : [11/15] 175375 - 175389<br>Domain: 180094 - 180594 [possible ORF:179518-181023, (IN (core))]<br>Domain: 181113 - 181451 [possible ORF:180954-181451, (RNase H)]	C	CCATC	0.0066	0.253846

	ATLANTYS_LTR	*Gypsy*	Location : 144468 - 153335 Len: 8868 Strand:+<br>5'-LTR : 144468 - 146046 Len: 1579 3'-LTR : 151758 - 153335 Len: 1578<br>5'-TG : TG , TG 3'-CA : CA , CA TSR : NOT FOUND<br>PBS : [14/16] 146075 - 146090 (CysGCA) GGCGGCAGCCCAAGAT<br>PPT : [11/15] 151663 - 151677 ACGATAGATTAGGGA<br>Domain: 150652 - 151053 [possible ORF:150040-151674, (RNase H)]<br>Domain: 151453 - 151671 [possible ORF:150040-151674, (IN (core))]	No	0.1356	5.21538

Table S4 Intact elements of DNA transposons across the *Sh4* region

Species	Family	Name	Position	Strand	TSD(+strand 5'-3')
O. sativa **(AA)**					
**DNA transposon**	OSTE30	Other	46781-47297	C	GTCTTGAC
	STOWAWAY8_OS	Stowaway	59189-59446	C	TA
	CLOUD-3	MuDR	64058-64610	C	CCTTTTTA
	TREP215	Stowaway	65663-65897	C	TA
	TREP215	Stowaway	71667-71905		TA
	STOWAWAY1_OS	Stowaway	89304-89431	C	TA
	CLOUD-7	MuDR	97827-98291	C	TTTTAAT
	TYPEU4	Other	133191-133583		5'_TAATGAATC 3'_CAATGAATC
	STOWAWAY10_OS	Stowaway	134462-134721		TA
	TYPEU	MuDR	150954-151310		T
	DITTO	Tourist	168321-168558	C	TTA
	STOWAWAY46_OS	Stowaway	177967-178114	C	TA
	STOWAWAY21_OS	Stowaway	189782-190017	C	TA
	TREP219	Stowaway	195439-195541		TA
	STOWAWAY1_OS	Stowaway	202010-202158	C	TA
	INDITTO	Tourist	202407-202671	C	TTA
	Gaijin	Tourist	203155-203297	C	TAA
	STOWAWAY43_OS	Stowaway	203418-203712		TA
	STOWAWAY21_OS	Stowaway	204256-204489		TA
	STOWAWAY18_OS	Stowaway	213683-213897	C	TA
	TYPEU4	Other	236145-236516	C	ATAGAAAAA
	STOWAWAY9_OS	Stowaway	237245-237474	C	TA
	OLO24	Tourist	240847-241189		5'_TGT 3'_TAT
	AMYLTP	Tourist	267093-267464		TTAGTTGCC
	STOWAWAY21_OS	Stowaway	267675-267908	C	TA
	OLO24	Tourist	268199-268517	C	CTT
	Gaijin	Tourist	270769-270915		AGA
	Gaijin	Tourist	278781-278927		ACA
	STOWAWAY50_OS	Stowaway	290204-290324		5'_CA 3'_TA
	STOWAWAY41_OS	Stowaway	293620-293879		TA
	Gaijin	Tourist	301973-302109	C	TAT
	STOWAWAY51_OS	Stowaway	302133-302364	C	NO
	CLOUD	MuDR	343091-343836	C	TAAATAATA
	TYPEG	MuDR	351168-351594	C	TTGTTATGA
	Explorer	Tourist	356055-356274		5'_TAC 3'_TAT
	INDITTO	Tourist	358224-358490	C	TTA
	CLOUD-4	MuDR	402553-403056	C	TTTAATTTA
	STOWAWAY1_OS	Stowaway	410693-410842		TA
	DITTO	Tourist	416896-417132		TAA

	STOWAWAY21_OS	Stowaway	424486-424717	C	TA
	UNIQUE	Tourist	427534-427679		TTG
	MPING	MITE/Tapo go	433777-434206		TTA
	Gaijin	Tourist	434987-435133		TTT
	STOWAWAY46_OS	Stowaway	435675-435826		TA
	STOWAWAY41_OS	Stowaway	437887-438137	C	TA
	TELIA	Other	441458-441598	C	TTTAAAAAA
	STOWAWAY9_OS	Stowaway	456839-457033	C	TA
	QIQI	Tourist	458610-458909		TAA
	STOWAWAY46_OS	Stowaway	474661-474783	C	TA
***O. punctata*(BB)**					
**DNA transposon**	STOWAWAY16_OS	Stowaway	35127-35350		5'_no 3'_TA
	TYPEU	MuDR	48732-49091	C	no
	STOWAWAY43_OS	Stowaway	76831-77062		TA
	ENSPM2_OS	En/Spm	118682-119377, 122096-122356	C	TAG
	STOWAWAY15-2_ OS	Stowaway	137614-137759		TA
	TREP220	Stowaway	159341-159462		TA
	Explorer	Tourist	165667-165886		no
	CLOUD-6	MuDR	167427-167916		TTATTTT
	STOWAWAY34_OS	Stowaway	196549-196805		TA
	TYPEU4	Other	211332-211711	C	5'_ACAGAGA 3'_ACAAAGA
	TYPEU4	Other	233204-233586		AT
	WANDERER_OS	Tourist	261085-261324	C	TTA
	QIQI	Tourist	313665-313965		5'_TTA 3'_TCA
	STOWAWAY46_OS	Stowaway	314787-314931		TA
	STOWAWAY41_OS	Stowaway	322735-322985		TA
	F804	Other	332325-332493		TATTTTATC
	TREP220	Stowaway	332713-332822	C	TA
***O. minuta* (BB)**					
**DNA transposon**	STOWAWAY43_OS	Stowaway	6527-6758		TA
	ENSPM2_OS	En/Spm	49479-50147,52 948-53209	C	?
	STOWAWAY46_OS	Stowaway	68261-68408	C	TA
	TREP220	Stowaway	90056-90177		TA
	Explorer	Tourist	96728-96946		5'_CTC_3'_CTA
	CLOUD-6	MuDR	98495-99014		TTATTTT
	STOWAWAY34_OS	Stowaway	127311-127567		TA
	TYPEU4	Other	175018-175401		AT
	WANDERER_OS	Tourist	183491-183731	C	TAA
	OSTE24	Other	201085-201709	C	5'_CGAGCG_3'_CGAGGG

	Gaijin	Tourist	211812-211950	C	ATA
	WANDERER_OS	Tourist	213140-213380	C	TTA
	STOWAWAY41_OS	Stowaway	218561-218825	C	TA
	DITTO3	Tourist	229668-229905		5'_TAA_3'_no
	STOWAWAY10_OS	Stowaway	239041-239294	C	5'_TA_3'_TG
	QIQI	Tourist	263045-263345		5'_TTA_3'_TTT
	STOWAWAY46_OS	Stowaway	264162-264316	C	TA
	STOWAWAY41_OS	Stowaway	272054-272305		TA
	F804	Other	281610-281776		TATTTTATC
	TREP220	Stowaway	281998-282107	C	TA
	Gaijin	Tourist	326852-326990	C	no
	STOWAWAY47_OS	Stowaway	350011-350147	C	TA
***O. minuta* (CC)**					
**DNA transposon**	TESS	hAT	18347-18570	C	GTTAGCAC
	TREP219	Stowaway	46207-46303		TA
	STOWAWAY41_OS	Stowaway	58958-59218	C	TA
	TYPEU4	Other	93334-93711	C	ATAGAAAAA
	STOWAWAY46_OS	Stowaway	105114-105280	C	5_TA_3_TG
	STOWAWAY18_OS	Stowaway	304496-304711		5_TG_3_TA
	TNR2A	Other	308362-308516		5_CGGTTTCAACTTCGGGG_3_TCTCTTCAACTTGAGGG
	STOWAWAY47_OS	Stowaway	308795-308930	C	TA
	OSTE23	Other	316063-316257	C	5_AAATCAAACAGCTAGCTATAGGAAGGATCAATGGTGAGCATGCATTTGGTTTTTTTTGC_3_AAGATGACTGTATATGAAAGCAATTAAATTAGTCATGACCCTGTTCTTCGTTTCTTTGC
	STOWAWAY41_OS	Stowaway	318492-318749		TA
	STOWAWAY47_OS	Stowaway	320371-320517		TA
	MUDRN1_OS	MuDR	338116-338852	C	5_GTATATTTTCA_3_GTATACATTCT
	STOWAWAY42_OS	Stowaway	340486-340728		TA
	MDM2	MuDR	349525-349818	C	TTAATTTTA
***O. officinalis*(CC)**					
**DNA transposon**	TESS	hAT	20622-20845	C	GTTAGCAC
	TREP219	Stowaway	42035-42130		TA
	STOWAWAY41_OS	Stowaway	64197-64456	C	TA
	TYPEU4	Other	99588-99965	C	ATAGAAAAA
	TYPEU4	Other	107586-107967	C	5_ATGGAAAAA_3_ATAGAAAAA
	STOWAWAY46_OS	Stowaway	118131-118297	C	5_TA_3_TG
	WANDERER_OS	Tourist	256304-256538		TTA
	STOWAWAY34_OS	Stowaway	272507-272775	C	TA
	STOWAWAY18_OS	Stowaway	375026-375240		TA
	TNR2A	Other	379214-379368		5_CGGTTTCAACTTCGGGG_3_TCTCTTCAACTTGAGGG

STOWAWAY47_OS	Stowaway	379644-379779	C	TA
OSTE23	Other	387783-387977	C	5_AAATCAAACAGCTAGCTATAGGAAGGATCAATGGTGAGCATGCATTTGGTTTTTTTGC_3_AGATGACTGTATATGAAAGCAATTAAATTAGTCATGACCCTGTTCTTCGTTTCTTTGC
STOWAWAY41_OS	Stowaway	391305-391560		TA
STOWAWAY47_OS	Stowaway	393192-393338		TA
MUDRN1_OS	MuDR	410706-411443	C	5_GTATATTTTCA_3_GTATACATTCT
STOWAWAY42_OS	Stowaway	413022-413264		TA

Table S5 Pair-wise relative rate test of duplicated genes between *O. minuta* and its diploid progenitors

Gene	BBvsBBCC_BB		CCvsBBCC_CC		BBvsCC		BBCC_BBvsBBCC_CC	
	$\chi 2$	P value	$\chi 2$	P value	$\chi 2$	P value	$\chi 2$	P value
**17**	0.33	0.5637	1	0.31731	0.33	0.5637	0.33	0.5637
**18**	2.67	0.10247	0.2	0.65472	3.52	0.06057	0.62	0.43277
**19**	2	0.1573	-	-	0.14	0.70546	1.8	0.17971
**21-1**	-	-	1	0.31731	0.14	0.70546	0	1
**21-2**	1	0.31731	0	1	1	0.31731	0.33	0.5637
**22**	-	-	ψ	ψ	11.84	0.00058	ψ	ψ
**23**	-	-	-	-	1	0.31731	1	0.31731
**24**	3	0.08326	2	0.1573	0.11	0.73888	1	0.31731
**25**	-	-	-	-	-	-	-	-
**26**	0	1	ψ	ψ	-	-	ψ	ψ
**29-1**	0	1	-	-	1.33	0.24821	1.33	0.24821
**29-2**	2	0.1573	-	-	1.8	0.17971	-	-
**29-3**	1	0.31731	0.33	0.5637	1	0.31731	0.67	0.41422
**29-4***	-	-	-	-	4	0.0455	4	0.0455
**30**	1	0.31731	0.33	0.5637	0	1	0.09	0.76302
**31**	0	1	1	0.31731	0.4	0.52709	1	0.31731
**32**	-	-	1	0.31731	3.6	0.05778	2.78	0.09558
**33**	-	-	0	1	1	0.31731	1	0.31731
**34**	2	0.1573	-	-	0	1	0.5	0.4795
**35**	-	-	-	-	0	1	-	-
**36***	5	0.02535	-	-	0.67	0.41422	4.45	0.03481
**38-1**	1	0.31731	-	-	0.4	0.52709	2.78	0.09558

**38-2**	0	1	1	0.31731	0.29	0.59298	0.6	0.43858
**39-1**	-	-	2.67	0.10247	5.56	0.01842	2.57	0.10881
**40**	2	0.1573	1	0.31731	0.33	0.5637	2	0.1573
**41**	0	1	1	0.31731	1.19	0.27523	1.8	0.17971
**42**	2	0.1573	1	0.31731	0.09	0.76302	0.08	0.78151
**44**	0	1	0.33	0.5637	0.06	0.80837	0	1
**45**	-	-	1	0.31731	0.33	0.5637	0	1
**46**	1	0.31731	-	-	0.08	0.78151	0.11	0.73888
**47***	1	0.31731	15	0.00011	0.6	0.43858	3.9	0.04819
**48**	1	0.31731	0.2	0.65472	2.78	0.09558	3.6	0.05778
**49**	1	0.31731	0	1	0.14	0.70546	0.67	0.41422
**50-1**	-	-	2	0.1573	0.2	0.65472	0.2	0.65472
**50-2**	1	0.31731	1	0.31731	0	1	0.25	0.61708
**51**	0.33	0.5637	0.33	0.5637	0.07	0.79625	0.6	0.43858
**52**	4	0.0455	0.11	0.73888	10.9	0.29715	0	1
**53**	2	0.1573	3	0.08326	0	1	0.09	0.76302

Note: "ψ" represents pseudogenes that were excluded from analysis; "-" indicates no estimation. *These genes have significantly different evolutionary rates, as revealed by pair-wise comparisons.

Table S6 Pair-wise estimates of Ka/Ks and divergence time among the BB, CC and BBCC genomes

Gene	BBCC_BBvsBBCC_CC		BBvsBBCC_BB		CCvsBBCC_CC		BBvsCC	
	**Ka/Ks***	**Divergent time (Mya)**	**Ka/Ks**	**Divergent time (Mya)**	**Ka/Ks**	**Divergent time (Mya)**	**Ka/Ks**	**Divergent time (Mya)**
**17**	0.2267	3.63846	1.0495	0.592307	0.3484	0.592308	0.2284	3.615385
**18**	0.0776	9.7	0.5937	0.269230	0.3934	0.546154	0.0729	9.584615
**19**	0.3392	3.26923	NA	NA	NA	NA	0.4768	3.269231
**21-1**	0.1798	6.26923	0	0.353846	0.2904	0.346154	0.1631	6.276923
**21-2**	0.1696	7.85385	NA	NA	0.4052	0.992308	0.1618	6.5
**22**	-	-	NA	NA	-	-	0.5555	6.715385
**23**	0.1492	7.66154	0	0.876923	NA	NA	0.1304	8.276923
**24**	0.2379	4.8	1.0054	0.207692	0.2255	0.638462	0.1413	5.092308
**25**	0	4.74615	0	0.761538	0	0.769231	0	4.761538
**26**	-	-	0	1.146153	-	-	-	-
**29-1**	0.7652	28.0308	-	-	0	2.823077	0.5775	16.48462
**29-2**	-	-	1.2386	1.284615	-	-	-	-
**29-3**	0.1757	18.2538	NA	NA	1.1854	1.330769	0.1296	16.37692
**29-4**	0.2938	13.2769	0.1969	2.407692	NA	NA	0.2609	12.91538
**30**	0.1287	7.1	0.6655	0.492307	0.2486	1	0.0974	6.807692
**31**	0.0562	8.65385	0.4201	0.515384	0.1039	0.515385	0.0654	8.253846
**32**	0.2273	6.38462	0	0.507692	0.1559	1.023077	0.3049	5.561538
**33**	0.1005	6.21538	0	0.669230	0.1477	1.307692	0.1426	4.384615
**34**	0.3013	5.71538	NA	NA	0	0.469231	0.2242	6.207692

**35**	NA	NA	NA	NA	NA	NA	NA	NA
**36**	0.1145	6.53846	1.5552	0.2	0.1548	0.4	0.0551	6.776923
**38-1**	0.2427	5.88462	0.3314	0.784615	NA	NA	0.1382	6.6
**38-2**	0.2978	7.56154	NA	NA	0.1632	0.9	0.3184	6.615385
**39-1**	0.1368	9.24615	0	0.261538	0.9059	0.523077	0.1656	9.569231
**40**	0.1659	4.21538	0.1343	2.584615	NA	NA	0.1919	5.215385
**41**	0.3349	8.94615	0.3415	0.884615	0.3438	0.661538	0.3291	8.846154
**42**	0.0798	11.2308	0.4116	0.707692	0.2073	0.7	0.0659	12.28462
**43**	-	-	0.2772	0.592307	-	-	0.4026	3.238462
**44**	0.3538	5.12308	0.6469	0.223076	0.4873	0.423077	0.3217	5.869231
**45**	0.1113	8.33846	0	0.976923	0.155	1.984615	0.1706	7.261538
**46**	-	-	NA	NA	-	-	0.1894	7.523077
**47**	-	-	0.3167	0.230769	-	-	0.135	6.569231
**48**	0.19	7.23846	0.1284	0.9	NA	NA	0.2195	7.846154
**49**	0.0786	10.8692	0.0853	1.423076	0.8837	0.592308	0.1181	10.72308
**50-1**	0.0327	10.7385	0	0.215384	0.6559	0.215385	0.0343	10.23077
**50-2**	0.2645	4.46923	0.1063	0.646153	0.1059	0.646154	0.2641	4.469231
**51**	0.6243	9.98462	NA	NA	0.6912	7.746154	0.1923	6.415385
**52**	0.1837	8.23077	0.7269	0.323076	0.3742	1.123077	0.1808	6.769231
**53**	0.0971	5.28462	0.2022	0.407692	0.1295	0.946154	0.0585	5.261538
	BC_BvsBC_C		BBvsBC_B		CCvsBC_C		BBvsCC	
**Average value**	0.2156	8.23803	0.4015	0.871203	0.3734	1.065659	0.20233	7.476923

Ka/Ks: If structural variation existed in the coding sequences between gene pair, we excluded this gene pair from the Ka and Ks calculation (indicated by "-"); "NA" indicates a Ks value of 0.

Table S7 Expression divergence of duplicated genes in allotetraploid *O. minuta*

	Gene code	BB-bias	CC-bias	Both transcription	Failure amplification
	17		√		
	18			√	
	19		√		
	21-1				F
	21-2	√			
	22			√	
	23			√	
	24	√			
	25			√	
	26			√	
	30				F
	31			√	
	32			√	
	33			√	
	34		√		
	35				F
	36			√	
	38-1			√	
	38-2	√			
	39-1			√	
	40			√	
	42			√	
	43	√			
	44				F
	45			√	
	46			√	
	47			√	
	48				F
	49	√			
	50-1			√	
	50-2			√	
	51			√	
	52	√			
	53			√	
**Total analysis genes**	34	6	3	20	5

Note: "√" indicates gene expression; "F" indicates failure in cDNA-SSCP analysis.

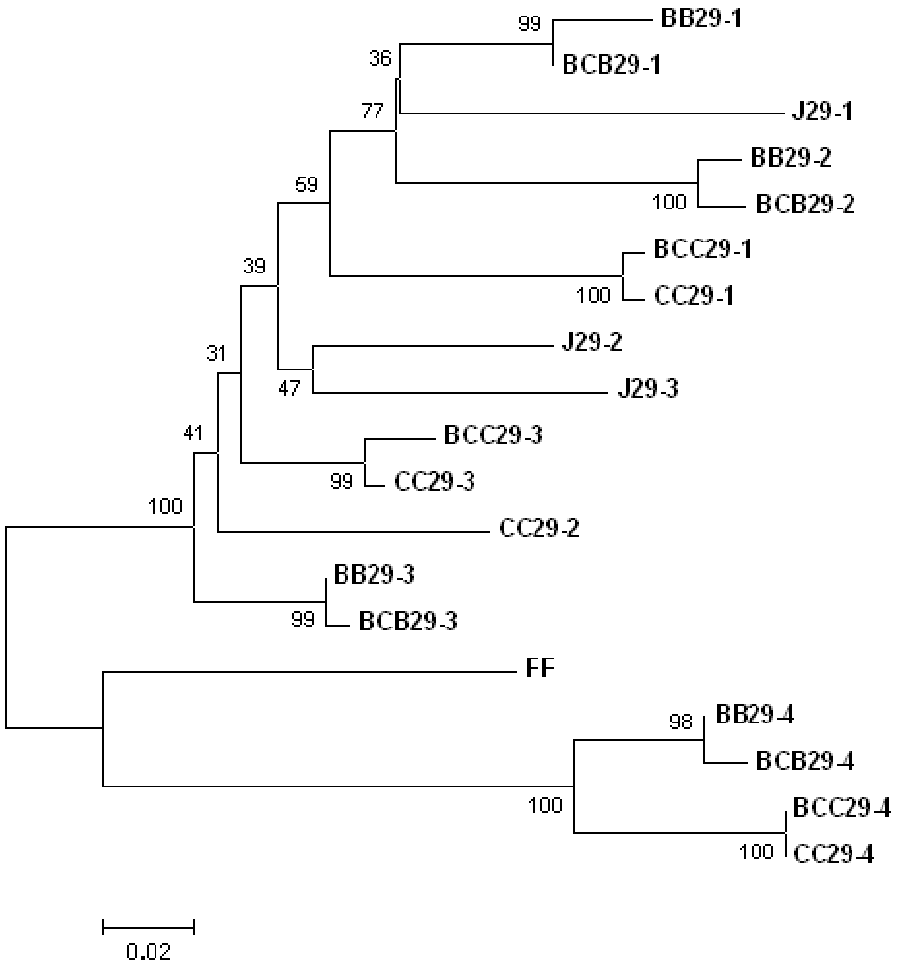

Figure S1 Phylogenetic analysis of Gene 29 clusters. Gene 29-4 in the BB, BBCC and CC genomes are clustered with FF gene 29, which is the only one in the FF genome. This result suggests that 29-4 is an ancient copy. After amplification, this copy was lost in the AA genome. J represents the rice genome, BB represents *O. punctata*, BCB and BCC represent the BB and CC-subgenomes in *O. minuta* and CC represents the *O. officinalis* genome. The phylogenetic relationship was constructed by the neighbor joining method with 500 bootstraps in MEGA5.2.

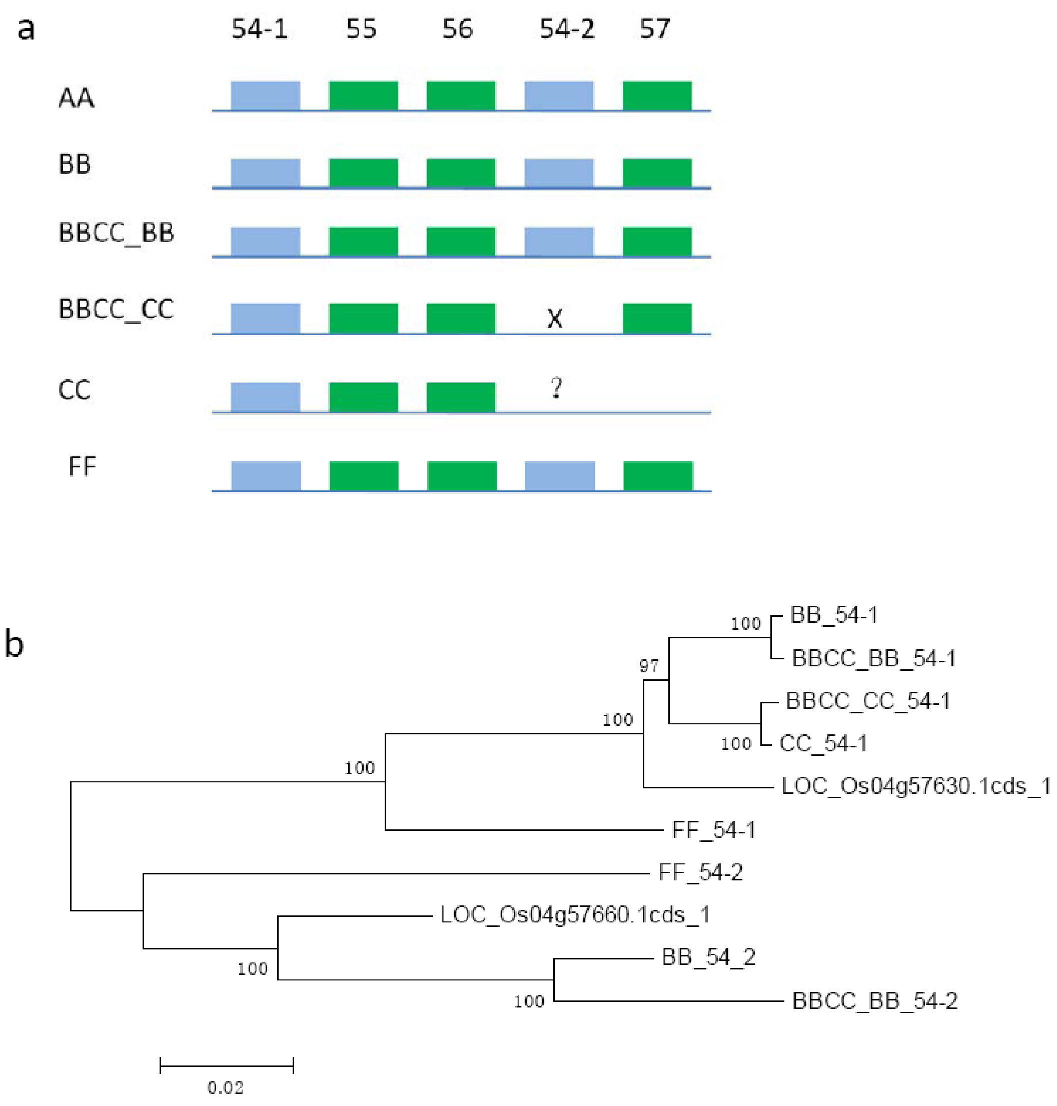

Figure S2 Evolutionary analysis of Gene 54 clusters. a) Gene content is well-conserved among *Oryza* species. "X" indicates a deletion and "?" indicates no sequence information. BB sequences were extracted from the unpublished draft sequence of *O. punctata*. b) The phylogenetic tree was built with the Neighbor-Joining method implemented in MEGA 5.2. *Japonica* genes are 57630(54-1) and 57660 (54-2).

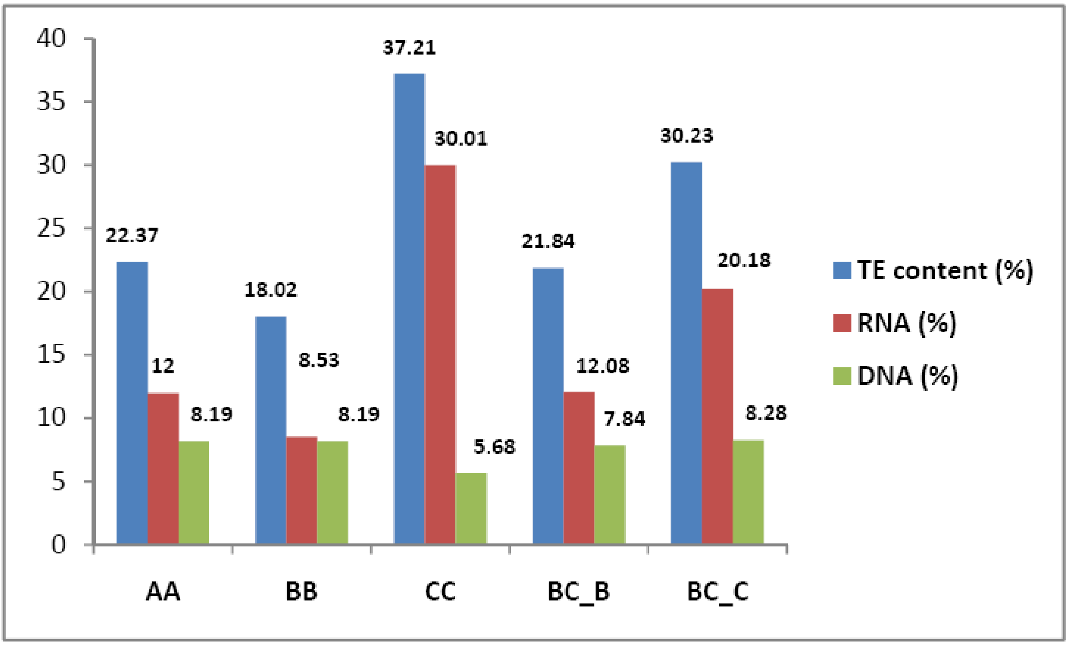

Figure S3 TE contents in five *Oryza* species. AA: *O. sativa* ssp. *japonica*, BB: *O. punctata*, CC: *O. officinalis*, BC_B: BB subgenome in *O. minuta* and BC_C: CC subgenome in *O. minuta*. RNA: RNA retrotransposon content, DNA: DNA transposon content and TE content: total transposon content.

(a)

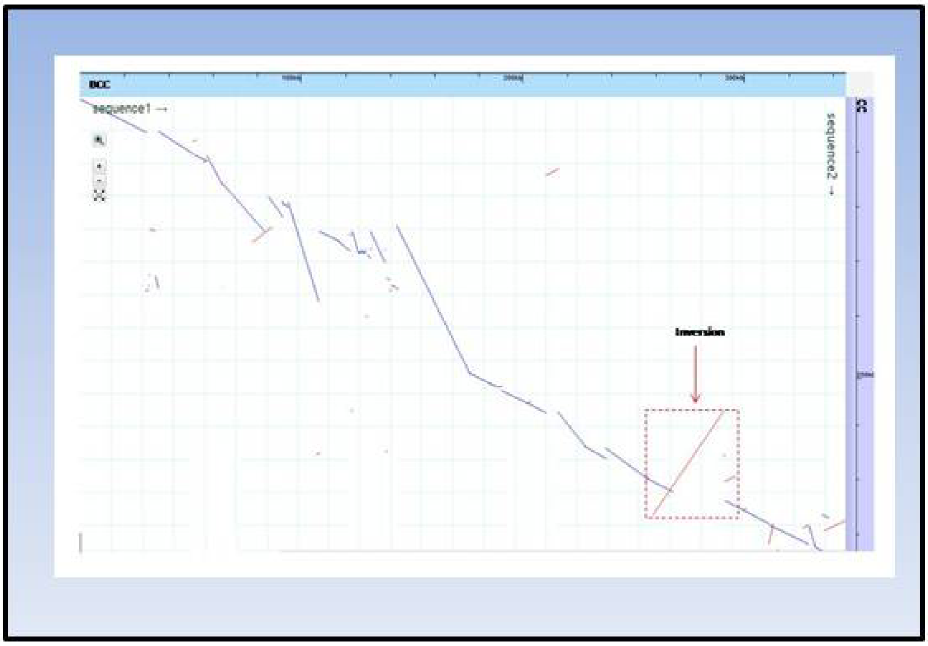

(b)

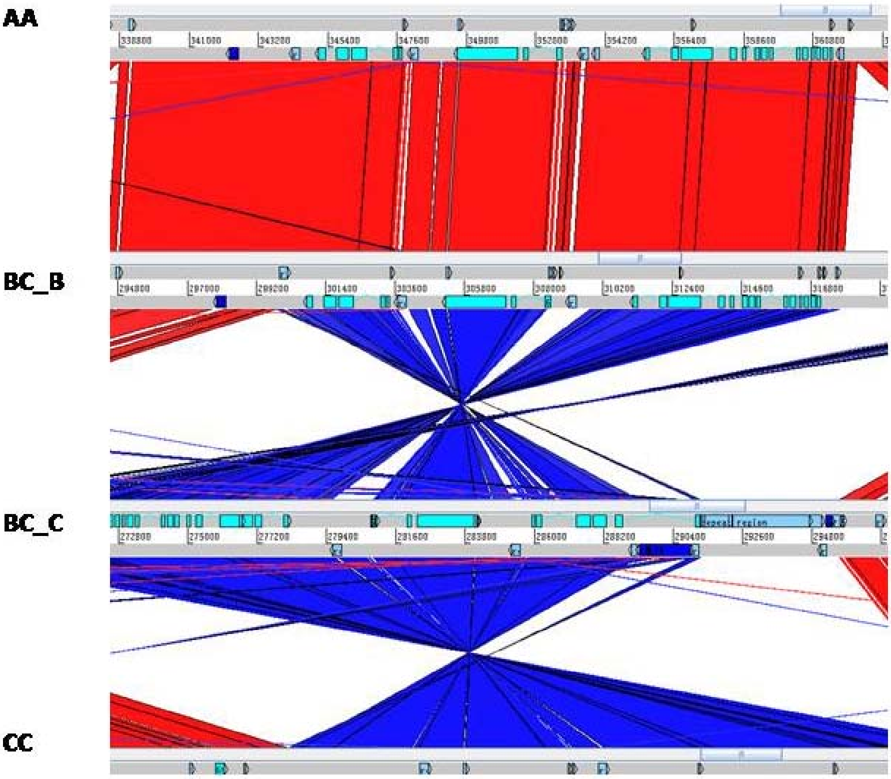

Figure S4 Large inversion detected in the CC subgenome of *O. minuta*. Comparative genomic analysis revealed a ~40 kb inversion containing three genes. The results were obtained using two independent approaches; (a) shuffle-LAGEN results and (b) ACT results.

(a)

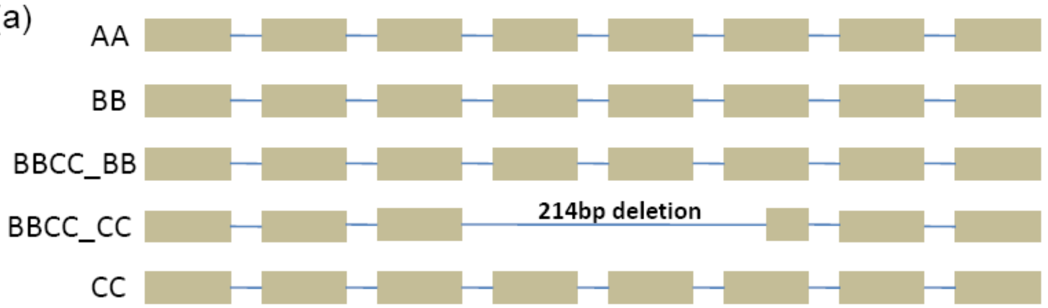

>LOC_Os04g57190
MAAVPSAAAPPVPVSPGERWPPLESSPDVFNQLMWSLGVPEDVAEFHDVYSLDADAL
EMVPQPVLAVVFCFPDPTQDASNPSQHLLITGEKETLFFIKQIESLGNACGTIALLHAVGN
AYSEISLSENSFLDMFIKSTSGMTSYERAVFLEKDDDMARAHLSAASAGDTKLSDDVEEH
YICFVECDGTLYELDGMKPGPINHGPSSSKSLLQVLQFCELGVAAVMSQNRT*

>BBCC_CC_22
MAALSTSSAAPAPVSPDERWPPLESSPDVFNQFMWSLGVPEDVAEFHDVYSLDADALE
MVPQPVLAVVFCFPDPTQDARNPSQHVLVTGEKLPVLVTQSYLTTLKSIIYVLSNVMEHS
MSLMV*SLDQ*IMACHLLKAYCRYRDFVNWVLQLSCL*IEH?

(b)

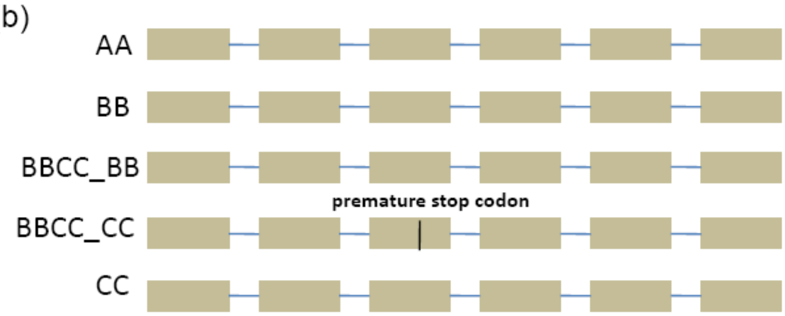

>LOC_Os04g57230.2 .
MAAAAAMAASRRLLHLRPELGLRLRSRALIPYPSWTKAMGRCRYEPSPRRLSSCRRAKNE
RPWRSSSNEQLTDGDGTAKYMGRNFPDEFAQLSLEEEESDDEVHGISETMVKDVEKAAVE
LLAGRAFTVSDLRKKLRAKKFPDNAVDSVIADFKSRGLLNDGYYAESFSRSRWLSSTWGP
KRIKQALRQKGVQDAEVDQATRRVFQDGHSNQTMYGISEDSMDHLFEQAAKQWQRGQSLP
LENRRARVVRWLQYRGFNWAVTNAIVRKLEAQKPP*

>BBCC_CC_26 .
MAAAAMAASRRLLHLRPKLGLCLRSRALIPSASWTNGMGRFRYEPSPRRLSSCRRAAKNE
RPWWSSSDEQLTHGDDGDGTAKYIGKNFPDEFAHLSLEEEETTMRCAESAKLW*KMWRRL
RSSCLLAEHSQFQTLGRNYVLRSFLIMQLIL*LLISSQGVC*MIVIMRNLFHDLDGYHQL
GTQSV*NRHFAKKGCKMQK*TRRQGEYSKMVIQTRQCMASQRIQWIISLSRQLNSGSGAK
ACPLRIAVHA**GGFSTEGSTGL*RMPLSGSWRHSNLP

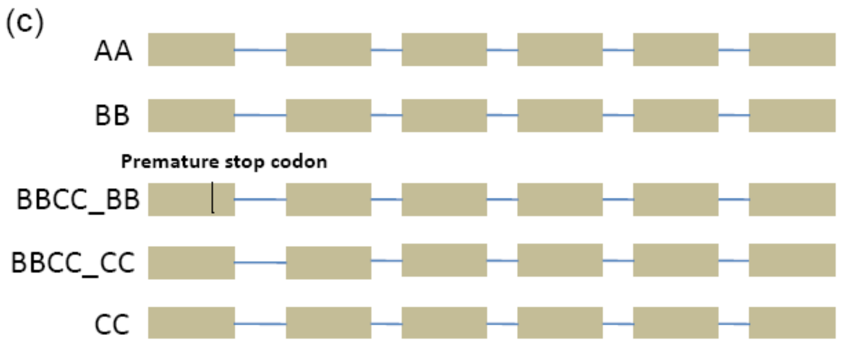

>LOC_Os04g57390.1
MEPATSRAPRSRFVVWLHGLGDTGRANEFLADSFPTTAAFADARWAFPTAPTAPVTCNRGMLMPSW
FDIHDAPITSVSVRDEEDVLRAVQSVHAMIDREIAAGTNPQDVFVFGLSQGGALGIASVLLHPKTLGGC
AVFSGFLPFNSSFAVRVTAQAKKLQCGLQTPVLWIHGQAGSLIPIKEGRDGIKFLRGLGMSCEFKVYDRL
GHSLEYYELDYCQRWVEKILHRSGREGLIRRVSRNIFLCSNLFNSS
>BBCC_BB_38
MEPTTSRAPVPLRGVAARPR*HRPRQRVPRRQLPHHHHGRRLRRRPLGLPHRAHHSDHLQPRHAHAL
VVRHPRRAHHFRYAGICKGRGGCTESCPKRARDDRRGYRGRDESARRVRFWT+PGRCVGHSKRAALP
ENFGRMCGIQWLPSL#FVFCCQSHRTSKEATICLADTRFVDPWATDSLTPIGEGRDGTKFLRGLGMSCE
FKVYDRLGHSLEYYELDYCQRWVDKILRRSRREGLVRWVSRNICLCNNLFNCSYHF

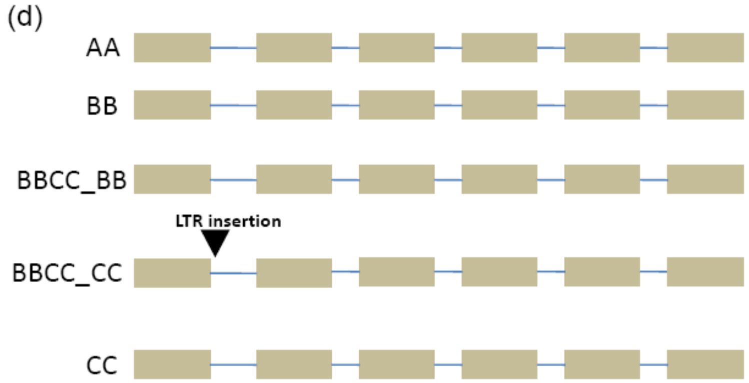

>LOC_Os04g57480.2
MARNKKKVAVAAPKARKPKRDAEEKKFAKKADMTEFRAQLDSLGLKIIELEGNEEEHMKYRAMIVQYIKEHRVDFEPFIEDEEPFEKYCDS
MLEDGTWAGHMELQAASILKRKNICIHMLNSPRWYIRNFSDREATSMIHLSYHQGEHYNSVRLREDPCQGPAMPVIIKADANVASTSNN
AQTKAKDLKKSSDRSKYDHISVKLVMAGTGCSNIAAVEQVLKDMDGDIDAAIEYMLAEQLILGSDDADGDPYLDYACDAEYVQTIEDELSM
KQDESQLDEHKKEEKDCSSKGETAQKHNSHSKKGKSKTKECSCGSARKHKPSCNLATTVASREPPKTTAPSREPPKTKGGQGKGQKGKKQK
KKEQDETPAIRDHDSKVAPDLGALCI
>BBCC_CC_43
MARNKKKVAVAAPKARKPKRDAEEKKFGKKADMTEFRAQLDSLGLKIIEVSADGNCFFRAMGDQLEGNEEEHIKYRAMIVQYIKEHRVDF
EPFIEDEEPFEKIL*LYD*GWDLGWSYGVASSFYSHKKKHMHLHA#LTTMVHKNFF*S*SYQYDSFIVSSE*ALQ+CQTEGRSMSRSCNADY
YQGRCQCS+HKQ#CSNKSKGLEEIFRQIKV*SYIG#IGDGWNWMF#CRCS*TGFERHGW*R*CCYRVHVS*ATNIGFQ*CGWRSLSGLC
M**VYTDHRG*V+HEAG*ESTG*AQERRKRLF+QRRNSSQTQQFTF#KGKVKNQGMLLWIC+EAQAFL#FSHDCSIKRTSKNNCTIERTSK
DQRWPRERPEGEEAKEERTS*NTSHPGS*F#SCTRSRSSLHM

Figure S5 Pseudogene annotation. The comparison for gene structures among five *Oryza* genomes and amino acid sequences for normal (*japonica*) and truncated products (pseudogene) for each set of orthologs were presented. (a) Gene 22: There is a 214 bp deletion in the CDS region in the BBCC_CC genome, which caused this gene to become a pseudogene. (b) Gene 26: An single-base-pair deletion occurred in the third exon of gene

26 in the CC subgenome of *O. minuta*. (c) Gene 38-3: A two-base-pair deletion occurred in gene 38-3 in the BB subgenome of *O. minuta*, which led to a premature stop codon. (4) Gene 43: An LTR retrotransposon was inserted near the first exon of gene 43 in the CC subgenome of *O. minuta*. RT-PCR and cDNA-SSCP experiments revealed this copy is not expressed. All sequences variations were reexamined and validated against the original Sanger sequences.

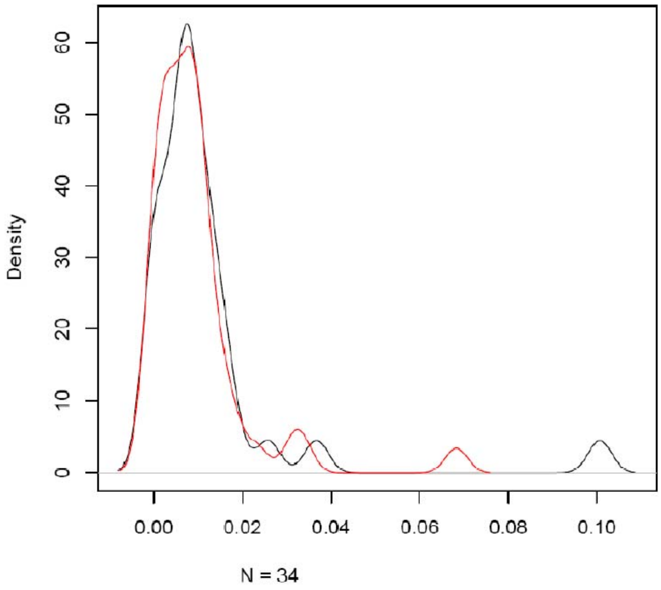

Figure S6 Distributions of Ks density for homoeologous genes between two subgenomes in *O. minuta* and corresponding diploid genomes. Most Ks are estimated to be around 0.01, and BB- and CC-type genomes show similar distribution patterns. Red line represents BC_B vs. BB and black line represents BC_C vs. CC. X-axis indicates Ks value, Y-axis indicates the density of the Ks value. N indicates gene number (which represents the genes used in the Ks calculation after excluding pseudogenes and genes with great structural variation).

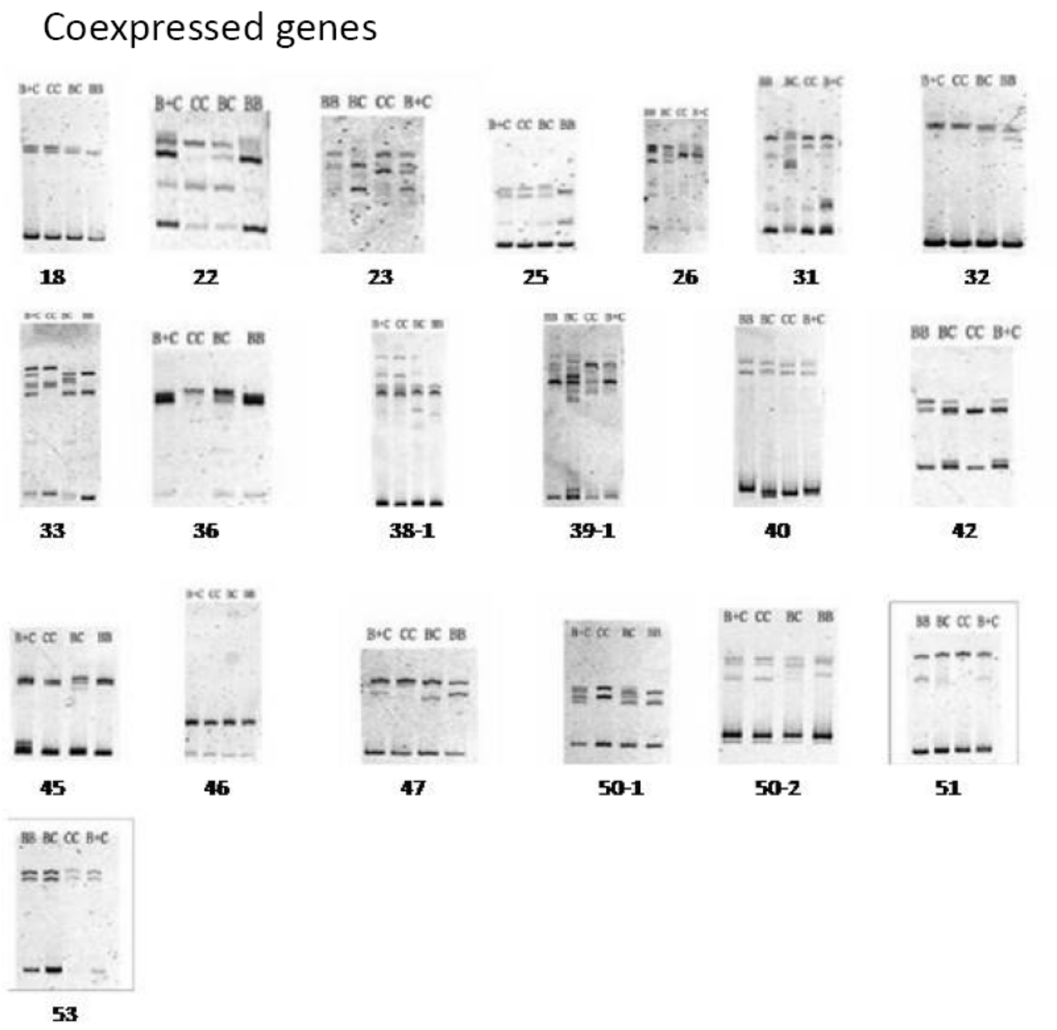

Figure S7 Results of cDNA-SSCP analysis with 34 pairs of duplicated genes. There were three types of cDNA-SSCP results: coexpressed, expressed in both BB and CC *O. minuta*; BC_B bias, BB copy expressed; and BC_C bias, CC copy expressed. The materials used to conduct cDNA-SSCP analysis included BB (*O. punctata*), CC (*O. officinalis*), BC (*O. minuta*) and B+C (mixture of *O. punctata* and *O. officinalis*).

BBCC_CC bias genes

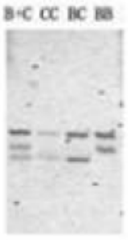

17

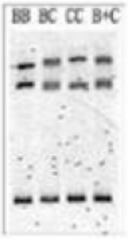

19

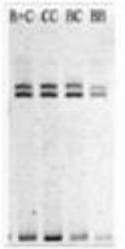

34

BBCC_BB bias genes

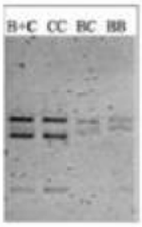

21-1

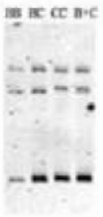

24

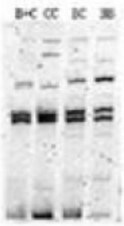

38-2

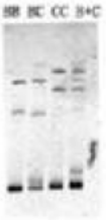

43

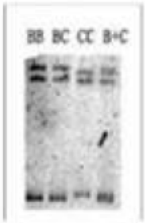

49

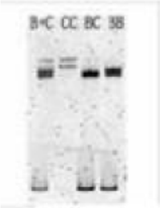

52

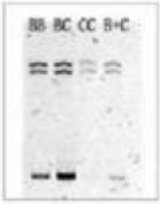

53

Figure S7 Continued
