# Supplementary material for: Genomic, regulatory and epigenetic mechanisms underlying duplicated gene evolution in the natural allotetraploid Oryza minuta
Source: BMC Genomics. 2014 Jan 6;15:11. doi: 10.1186/1471-2164-15-11 (PMC3890553; doi:10.1186/1471-2164-15-11)
Supplement: Additional file 2 — Primer information in this study. [file 1471-2164-15-11-S2.pdf]

Table S1 Primer information in this study.

TIGR V6 name (LOC_OS04g)	Gene number	Primer name	Sequence (5'-3')	Length (bp)	Tm(℃)
**For cDNA-SSCP**					
57130	17	s130-F	CCGCTGCTCGGTTTCCTC	161	62
		s130-R	CGAGTTCCGGTGCAGGCT		
57140	18	s140-1-F	GTCTCACCAGCAGAGTCCAAC	182	55
		s140-1-R	TCCAGGTCGTCGTCTTCACTA		
57150	19	s150-F	GATGGATGACCCGCTTATGT	218	55
		s150-R	TTTCCCAGTCTCCAGTGCTA		
57180	21-2	S180-F	CACCAACTTACAGGGAGAAA	217	50
		S180-R	CCATAATCAGAGTGAGTAGCC		
57190	22	S190F	CTCGAGTCGAGCCCCGACGT	143	61
		S190R	CTGGGTTGGATCGGGGAAGC		
57200	23	S200-F	CCAAAAGCTGACGGTGGTCG	Jap:294	58
		S200-R	CAGCAGATGACGCACGAGTT	Other<294	
57210	24	S210-F	AGAAAGCAATACAAGGACAC	231	51
		S210-R	AAATCCAGTAAGTTTGAGGG		
57220	25	S220-F	CAAAGGTCCTCCTGTCAAT	118	51
		S220-R	CGAGCGGTGGATTCATACTT		
57230	26	230-SS&STR-F	CTCCCGTGCCCTAATCCCCT	290	64
		230-SS&STR-R	CAGCCTTCTCCACATCTTTCACCA		
57300	31	S300-F	GCT GGA GGT TGT CTG CAT GG	287	52
		S300-R	GGA CAC GCT CCG ATG AAC GC		
57310	32	S310-F	GGGAAAGAAACAAATCCTATG	199	50
		S310-R	GCATAAATCCAACCAAGTC		
57320	33	S320-F	GGT GGC CGT CGA GGA GTC CT	196	60
		S320-R	GCG TAG TTG CGG TCA CGG TA		
57330	34	S330-F	GCGACACCTTCTTCTCCACC	287	55
		S330-R	CCACCCTAGCGCATACCC		
57350	36	S350-F	TTCGTGAAGCGTTCGAGGTC	290	55
		S350-R	AGATCGGGGATGTCGTAGGC		
57370	38-1	E57370-F	AACGAGCCCATCCGCAACT	355	62
		E57370-R	AACCGAAAGGCACCCACCC		
57380	38-2	E57380-F	TTCCTATTACTGCTAGAACTGC	287	52
		S57380-R	CATGCCATGAAACCACAAAAC		
57400	39-1	E57400-F	CGGCAACCTCAACTTCGTCT	311	60
		E57400-R	CCATGTAATCCGCCATGTGC		
57420	40	E57420-F	CACTCCCTAAACTAGAGGTCTCA	314	55
		E57420-R	AATGATGGTATCTAAGTGCCTTG		
57440	42	E57440-F	GCTGTCGGCTATGGCACT	164	55
		E57440-R	CCGCTCTTGGTAGGGTATG		

57480	43	E57480-F	CGGTTAAATTGGTGATGG	392	50
		E57480-R	TGCTACAGTCGTGGCTAA		
57500	45	E57500-F	GATGAGTGGTGGTGATGG	282	50
		E57500-R	GCCAAAGGAACGGAGATA		
57510	46	E57510-F	TTCAAGGGAGCCAAACAC	210	53
		E57510-R	AGCAACGGCATCAAAGAG		
57520	47	S520F	TTCCTGCCATTCTTCTGATT	195	54
		S520R	TTTTACCTTGTTTGCCGATA		
57540	33	E57540-F	GCTTGAAGAGGGAGCAGAA	129	53
		E57540-R	TCTCGGAGTTTGAGGTTGA		
57550	34-1	S550F	AATCAAAGGGTAACCAAAAT	236	50
		S550R	TTAGGCCAGAAGACTGTATC		
57560	34-2	S560F	ATTCGCTTGGTTCTTATTCC	245	53
		S560R	CATCTCCTCCCTCATGATAAT		
57590	35	E57590-F	GGAGTAGTCCAGAACCGTAA	213	51
		E57590-R	TATCCGTTTGTTTGAGCC		
57600	36	E57600-F	TCCGAGAAAGAGCGAGAT	206	52
		E57600-R	CGATGAGGGAAGTTGTGC		
57610	37	S610F	ACAGCCGTTTCACTATTTTC	181	50
		S610R	CTAACTTCAGTTATCGTCCC		
**For methylation analysis**					
150	19	BB-1-F	TGGGAATTTGTAGTTTATGTTTAGATG	331	
		BB-1-R	ACTAATCCAATAAACCCATCCTATC		
		BB-2-F	TGTTTTTGAGAGTATTGTTTTTTTT	226	
		BB-2-R	ATATAAACCCCATACCTTTTCCCT		
		BC_B-1-F	ATTATTAGTTTAGTTTATTATTTTTTTT	237	
		BC_B-1-R	ACTCATCCTATCAATTACTAAAATC		
		BC_B-2-F	GATAGGATGAGTTATAGGTGGTTAAA	572	
		BC_B-2-R	TCAACCTCTCTATCCCTCTAATTTATAA A		
		BC_C-1-F	TTTTAATGGGATAATTTTTAATGTA	378	
		BC_C-1-R	ACCCATCCTATCAATTACTAAAATC		
		BC_C-2-F	TTTATTTTATGTTGTTGTTTTTTTT	296	
		BC_C-2-R	AAAACCTAACCTCTAAATCCAAC		
		BC_C-3-F	TTTTTTTTGTTTTGAGTGTTGTGTT	345	
		BC_C-3-R	CCAACTAATATCTAATTACCATACCTA ATA		
		BC_C-4-F	GATTTGGTATTTATGGATATTAGGTTTG	316	
		BC_C-4-R	ATAAAACAAAAAAACACCTACCCAC		
		CC-1-F	GAGTTTAGGTGGTATAGATTAAGTTGA TG	218	
		CC-1-R	AAAAAACCTAACCTCTAAATCCAAC		
		CC-2-F	TTTATTTTATGTTGTTGTTTTTTTT	481	
		CC-2-R	AACTCACAACTAATACCTTATAAAC		
